# Targeting Pim kinases in hematological cancers: molecular and clinical review

**DOI:** 10.1186/s12943-023-01721-1

**Published:** 2023-01-25

**Authors:** Marcia Bellon, Christophe Nicot

**Affiliations:** Department of Pathology and Laboratory Medicine, Center for Viral Pathogenesis, University of Kansas Medical Center, 3901 Rainbow Boulevard, Kansas City, KS 66160 USA

**Keywords:** Pim1, Pim2, Pim3, Leukemia, Lymphoma, PIM, JAK, STAT, B-cell, T-cell

## Abstract

Decades of research has recognized a solid role for Pim kinases in lymphoproliferative disorders. Often up-regulated following JAK/STAT and tyrosine kinase receptor signaling, Pim kinases regulate cell proliferation, survival, metabolism, cellular trafficking and signaling. Targeting Pim kinases represents an interesting approach since knock-down of Pim kinases leads to non-fatal phenotypes in vivo suggesting clinical inhibition of Pim may have less side effects. In addition, the ATP binding site offers unique characteristics that can be used for the development of small inhibitors targeting one or all Pim isoforms. This review takes a closer look at Pim kinase expression and involvement in hematopoietic cancers. Current and past clinical trials and in vitro characterization of Pim kinase inhibitors are examined and future directions are discussed. Current studies suggest that Pim kinase inhibition may be most valuable when accompanied by multi-drug targeting therapy.

## Introduction

Pim kinases (*P*rovirus *I*ntegration site for *M*oloney leukemia virus) are a family of serine/threonine protein kinases with roles in cellular development, immunoregulation, and oncogenesis. Early studies of the original Pim kinase, Pim-1, led to the discovery of an oncogenic role for the Pim kinase family in lymphoma [1, 2]. Even though Pim-2 only shared 55% amino acid homology with Pim-1, it was considered a compensatory protein to Pim-1, as it shared a very similar kinase domain [3]. A third member of the family, Pim-3, was found to catalyze histone phosphorylation and autophosphorylation [4]. All three Pim proteins can phosphorylate serine and threonine amino acids [5]; and can activate similar cellular pathways. This has led researchers to believe that individual Pim kinases are compensatory, whereby the loss of one Pim kinase can be alleviated by the expression of another Pim kinase. There are several counterpoints to this theory, since individual Pim kinases have different expression patterns in cancer(s), distinct tissue locations, dissimilar regulation, and are encoded on different chromosomes [6]. Furthermore, Pim-1 and -2 have alternative isoforms that likely have unique functions. This suggests that while similar, individual Pim kinases may have distinct roles, which favor cellular distribution or various tumor microenvironments. This would explain why Pim-3 is highly over-expressed in some solid cancers, such as prostate and breast cancer, while Pim-1 and Pim-2 are generally over-expressed in hematopoietic cancers. The study of genetically modified mice deleted for Pim-1,-2, and-3 demonstrate that Pim kinases are not embryonically lethal, but exhibit reduced body sizes at birth and throughout life [7]; while mice that are knocked out for all three Pim kinases lack proper hematopoietic cell development and regulation [8]. Despite their expression in tumorigenic cells, Pim kinases are considered “weak” oncogenes since over-expression studies produce tumors at low frequency and after a long latency period. Pim kinases may have larger roles in tumor progression, rather than development; and have been proposed as broad drivers of chemotherapy resistance [9]. While an independent role in cancer initiation is weak, Pim kinases show especially strong synergistic roles in the presence of other oncogenes, such as c-Myc/n-Myc (C/N-MYC Proto-Oncogene), and Bcl-2 (BCL2 Apoptosis Regulator) and in the presence of various carcinogens to initiate cancer [10].

**Fig. 1:**
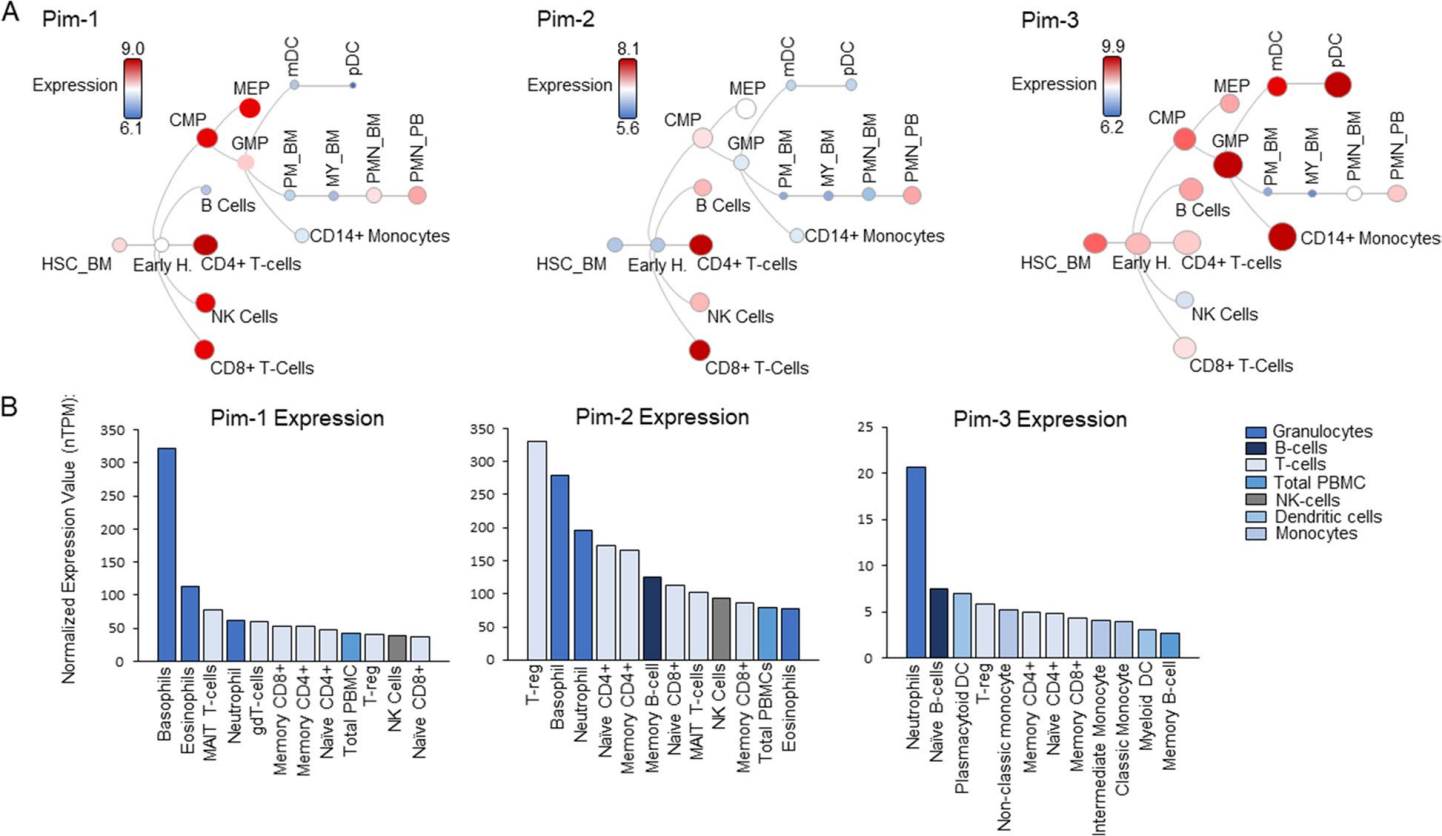
Pim kinase expression during normal hematopoiesis. **A** Hierarchical differentiation trees for the expression of Pim-1, − 2, and − 3 during normal hematopoietic stem cell differentiation from the bone marrow (HSC-BM). Pim kinase expression levels are indicated by the legend, whereby the size and color of the nodes indicates general expression patterns. Data are derived from Normal human hematopoiesis (HemaExplorer) obtained from the BloodSpot Data base (https://servers.binf.ku.dk/bloodspot/) [11]. Definitions are as follows: CMP (Common myeloid progenitor cell), GMP (Granulocyte monocyte progenitors), MEP (Megakaryocyte-erythroid progenitor cell), NK cells (CD56+ natural killer cells), PM_BM (Promyelocyte from bone marrow), MY_BM (Myelocyte from bone marrow), PMN_BM (Polymorphonuclear cells from bone marrow), and PMN_PB (Polymorphonuclear cells from peripheral blood). **B** Pim-1, − 2, and − 3 expression patterns in blood samples. Graphs represent the normalized transcript expression values, denoted by average TPM (transcript per million). Data are derived from HPA (Human protein atlas) RNA-seq from the Human Protein Atlas database (https://www.proteinatlas.org/)

The over-expression of Pim kinases is largely specific to hematologic cancers compared to solid tumors, though exceptions do exist, particularly regarding Pim-3. Generally, Pim kinases are expressed at higher levels in hematopoietic cells compared to other tissue. Expression data obtained during normal human hematopoiesis shows Pim-1 and Pim-2 have similar distribution patterns, with stronger expression in CD4 and CD8 cells (Fig. 1A) (https://servers.binf.ku.dk/bloodspot/) [11, 12]. Indeed, Pim-1 protein is expressed during normal embryonic development in hematopoietic tissue especially the liver, spleen, and bone marrow, but is shut off in most adult tissues [13, 14]. Pim-3 differs significantly, whereby expression is higher in granulocyte monocyte progenitors (Fig. 1A). Data obtained from the Human Protein Atlas (http://www.proteinatlas.org) and publications, also confirms low, non-specific gene expression of Pim-3 in various adult tissue [15]. This contrasts with Pim-1 and Pim-2 with higher expression in cells of the bone marrow/lymphoid and in blood cells (Fig. 1B). In conjunction with their cellar distribution, Pim-1 transgenic mice are susceptible to lymphomas; and Pim kinases have roles in the differentiation, activation, and/or response of immune cells [16, 17]. Given the role of Pim kinases in tumorigenesis and their over-representation in immune cells, it should come as no surprise that Pim kinases often display robust expression in hematopoietic cancers.

## The role of Pim kinases in cancer

Pim kinases phosphorylate proteins involved in pro-survival and key T- and B-cell signaling pathways, which contribute to Pim kinase transforming properties in leukemias and lymphomas. In vivo evidence by way of gene insertion, knock-out, and transgenic mouse studies largely established Pim kinase involvement in initiating tumorigenesis independently of other oncogenes or tumor suppressor inactivation. Insertion of the Moloney-murine leukemia virus (M-MuLV), into either Pim gene locus leads to enhanced protein expression of Pim-1/− 2 that can lead to T- and B-cell lymphomas [1, 3, 18]. The use of Pim-1 and Pim-2 expressing lymphoid cells in transgenic mice also causes a low frequency of lymphomas after long latency periods; whereas Pim-3 expressing liver cells of transgenic mice produce carcinomas but only in the presence of carcinogens [18]. Pim-1 transgenic mice are also more susceptible to accumulating mutation(s) in response to genotoxic agents’ exposure, as evidenced by an increase in tumor development in Pim-1 transgenic mice exposed to carcinogens or ionizing radiation [19, 20]. These in vivo models demonstrated that Pim kinases are oncogenic; however, studies have also shown that when expressed cooperatively with other oncogenes, tumorigenesis is significantly enhanced. In particular, the Myc pathway (including c-Myc, N-Myc, and L-Myc) is strongly associated with Pim kinase-mediated transformation; and the two pathways act synergistically in tumorigenesis. Myc is often overexpressed at the gene/protein level or by translocation or rearrangement in a broad group of leukemias and lymphomas [21]. The cooperation between Pim and myc in the generation of leukemia/lymphoma is well documented: Pim-1/myc transgenic mice develop pre-B-cell leukemia at high frequency [16], c-Myc/N-Myc enhances Pim-1 development of T-cell lymphoma in utero [22], and Pim-2/c-Myc mice develop pre-B-cell, B-cell, and T-cell lymphomas [23]. A role for Myc in cooperating with Pim-3 in the develop of B- or T-cell lymphomas/leukemias has only been shown indirectly [24]. However, in Myc transgenic mice harboring Pim-1/− 2 deletion, Pim-3 was activated in tumor cells [25]. Several loci that enhance Myc/Pim kinase tumor development have already been identified, including Pal-1/Gfi-1 (Growth Factor Independent 1 Transcriptional Repressor), Bmi-1 (B Lymphoma Mo-MLV Insertion Region 1 Homolog), and Runx2 (RUNX Family Transcription Factor 2) [18, 26, 27].

**Fig. 2:**
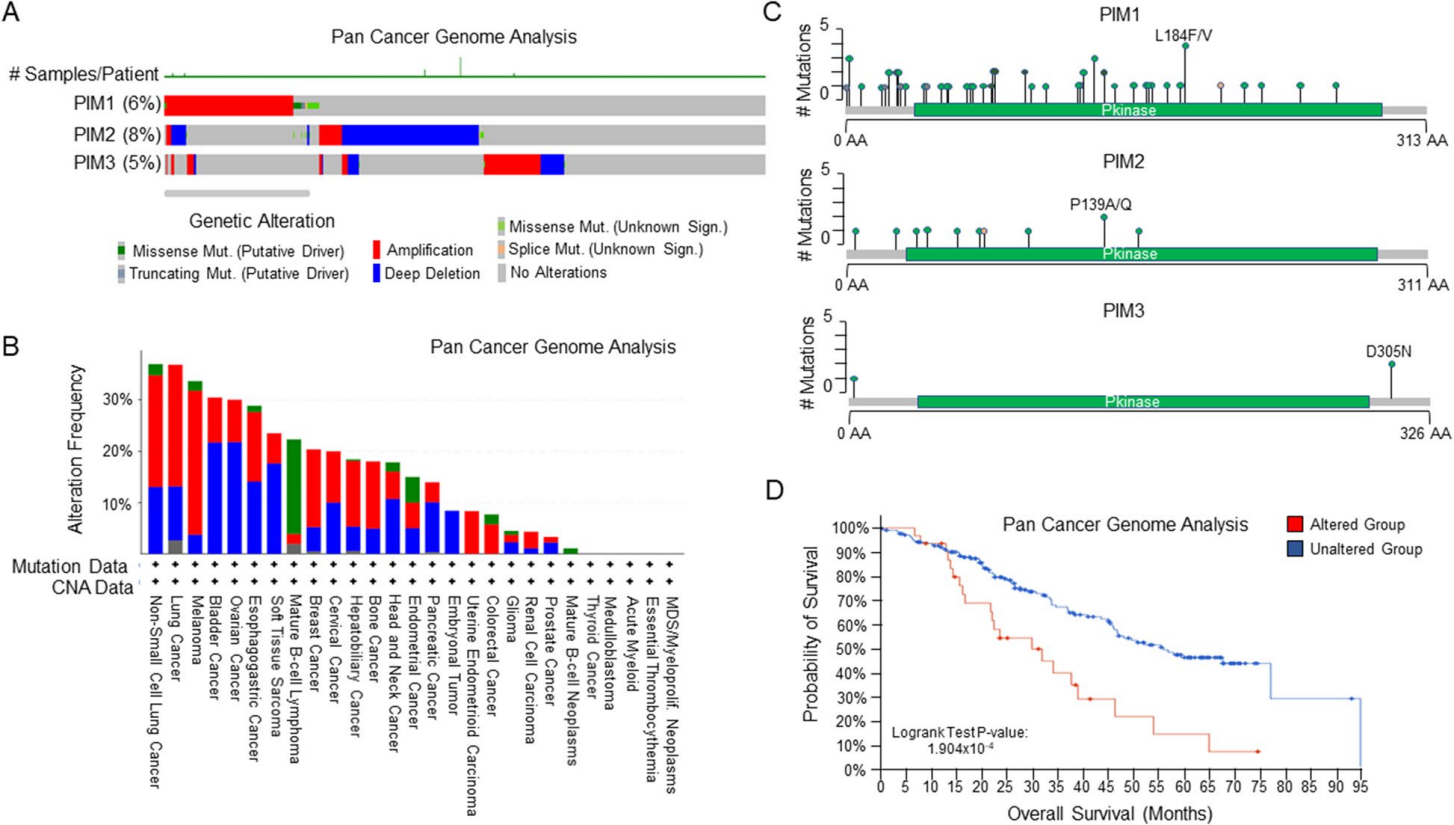
Pan Cancer Analysis of Genetic Alterations in Pim kinases. **A**-**D** Results derived from the PanCancer Analysis of whole genomes (https://www.cbioportal.org/) for Pim-1, − 2, and − 3. Data encompasses whole genome sequencing from 2583 whole cancer genomes and matched normal tissue across 38 tumor types [28, 29]. **A** Oncoprint of genetic alterations in Pim kinases. Missense mutations (both putative drivers and unknown significance), splice and truncating mutations, amplification and deep deletions are shown. **B** The frequency of cancers harboring mutations, amplifications, and deep deletions in the Pim kinases are shown. **C** Mutations in Pim-1, − 2, and − 3 are demonstrated. Most mutations in Pim-1 are derived from samples with follicular lymphoma, nodal marginal zone lymphoma, and DLBCL. The position of the mutations in the Pim gene and the frequency of the mutation are shown. The most common mutation in each of the Pim genes is noted (Pim1: L184F/N, Pim2: P139A/Q), and Pim3: D305N). **D** Patient overall survival in individuals harboring Pim genetic alterations. Data represent 36 patients with alterations in Pim genes and 245 patients with unaltered Pim genes

Pim kinases are constitutively active. They are largely overexpressed in cancer cells due to transcriptional activation and stabilization through positive feedback loops with upstream regulators, such as the JAK/STAT (Janus kinase/signal transducer and activator of transcription), PI3K/AKT (PI3-kinase/AKT serine/threonine kinase 1), and NF-κB (NF-Kappa-B transcription factor) signaling pathways. An examination of Pim kinase genomic alterations in cancer is largely lacking, even though mutations and gene modifications are prevalent. A Pan-cancer genomic analysis of Pim kinase mutations found numerous gene amplifications, deletions, missense mutations, and splice mutations in all 3 Pim kinases (Fig. 2A) [28–30]. Of note, this database did not include an analysis of non-solid tumor tissue; largely excluding leukemias and most lymphomas. Genetic alterations were found in 6, 8, and 5% of Pim1, Pim2, and Pim3, respectively, in the cancers examined. The most common alterations in the 3 kinases were gene amplifications and deep deletions (Fig. 2B). In contrast to other malignancies, mature B-cell lymphomas displayed a high rate (18.5%) of mutations, especially in Pim1 (Fig. 2C). The most common mutations were Pim1 missense mutations at L2F, L184F/N, and E135K. Data is still lacking on the biological significance of these mutations as it relates to Pim kinase activity and oncogenicity. However, due to their location in recurrent hotspots, most Pim1 mutations are hypothesized to be oncogenic. Examination of survival data suggests, but does not confirm, that alterations of the Pim genes decreases overall patient survival (Fig. 2D). These results will need to be confirmed in controlled, matched, and cancer specific patient samples.

**Fig. 3:**
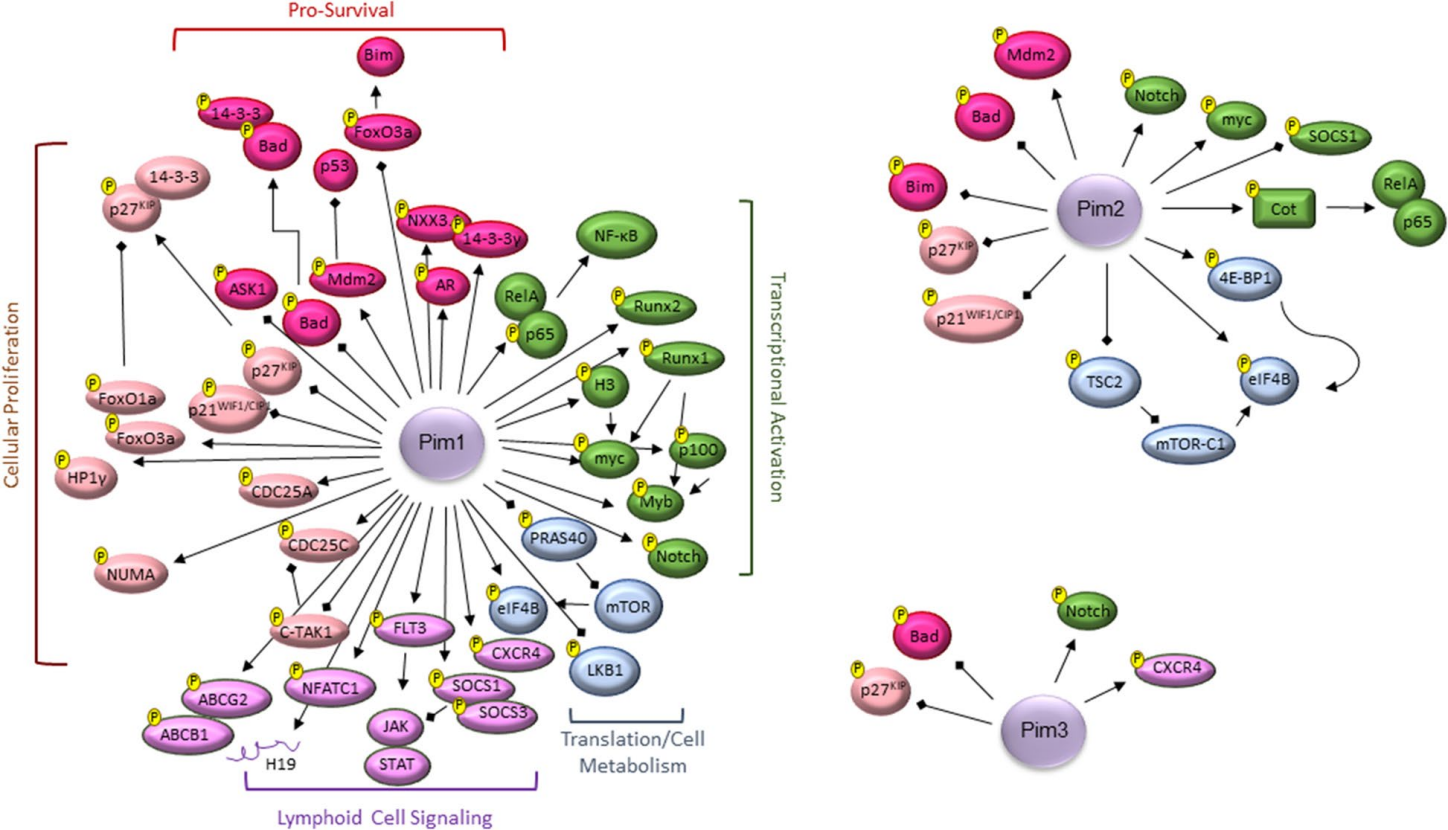
Current Pim-1, − 2, and − 3 Phosphorylated Cellular Targets. Cellular Pim kinase substrates are grouped based on their effect on transcriptional activation (green), translation/cellular metabolism (blue), survival (dark pink), proliferation (light pink), and lymphoid signaling (purple)

The pro-tumorigenic phenotype of Pim kinases is due to their phosphorylating specific serine/threonine motifs and increasing the activity of target proteins. These substrates have key roles in cell proliferation, cell survival, cap-dependent translation, metastasis, and tumorigenesis. The ability of Pim kinases to phosphorylate proteins involved in transmembrane drug efflux pumps, such as the ABC transporters (ATP-binding cassette transporters), increases their expression levels and leads to multi-drug resistance [31–33]. The multilateral effects of Pim kinases on translational components allow for increased microRNA (miRNA) function and polysome occupancy, allowing for miRNA targeting despite an enhanced need for protein synthesis in activated or transformed immune cells [34]. This effect was greater for JAK/STAT/Pim signaling than for the parallel, PI3K/AKT/mTOR (mammalian target of rapamycin) signaling pathway. Their role in regulating translation causes Pim kinases to directly affect the metabolic function of cells. Triple knock-out mice for all three Pim kinases demonstrate reduced growth, protein synthesis, and 5′-cap dependent translation [35]. While a detailed analysis of every target is beyond the scope of this review, a brief description of currently known targets is listed below (Table 1), not only to demonstrate the vast array of Pim kinase targets, but notably, to highlight the oncogenic and central role of Pim kinases in cancer (Fig. 3). While it may appear that Pim-3 does not have as large of a role in initiating and maintaining transformation of cells, this may be due to the lack of studies on Pim-3 phosphorylation targets, compared to the other Pim kinases.

**Table 1:** Pim Kinase phosphorylation substrates

	Direct Phosphorylation Targets	Effect on Oncogenesis	Pim(s)
**Transcriptional Activation**	**p65/RelA** [36]	NF-κB signaling pathway	Pim1
	**Cot** (MAP3K8/Mitogen-Activated Protein Kinase Kinase Kinase 8) [37]	NF-κB signaling pathway	Pim2
	**c-MyC** (myc Proto-Oncogene Protein) [38]	c-Myc and NF-κB Signaling pathways	Pim-1 and 2
	**H3** (Histone H3) [39]	Enhance c-Myc transcriptional Activity	Pim-1
	**C-MyB** and **A-Myb** (Myb Proto-Oncogene) [40]	Enhance transcriptional activities; Feedback: c-Myb can regulate c-Myc [41]	Pim-1
	**p100** (Nuclear Factor Kappa B Subunit 2) [42]	Up-regulates c-Myb; NF-κB signaling pathway	Pim-1
	**Runx1** and **Runx2** (Acute Myeloid Leukemia Gene 1 and 2) [43]	(CBF) family of transcription factors; Required for Myc and Myb activity in leukemia [44]	Pim-1
	**Notch1** and **Notch3** (Notch receptor 1 and 3) [45]	Transcriptional Activators (cleaved); activate c-Myc	Pim-1, −2, −3
**Proliferation and Cell Cycle**	**p21CIP1/WAF1** (CDKN1A/cyclin Dependent Kinase Inhibitor 1A) [46, 47]	Cell Cycle Progression	Pim-1 and 2
	**p27KIP1** (CDKN1B/cyclin Dependent Kinase Inhibitor 1B) [48]	Cell Cycle Progression	Pim-1, −2, −3
	**FoxO1a** and **FoxO3a** (Forkhead box O1a and 3a) [49, 50]	Cell Cycle Progression	Pim-1
	**C-TAK1** (protein kinase Cdc25 C-associated kinase 1) [49]	Cell Cycle Regulator	Pim-1
	**CDC25A** and **CDC25C** (Cell Division Cycle 25A and C) [50, 51]	Cell Cycle Progression	Pim-1
	**NuMA** (Nuclear Mitotic Apparatus) [52]	Mitosis	Pim-1
	**HP1γ** (heterochromatin-associated protein 1 gamma) [53]	Mitosis	Pim-1
**Translation and Cell Metabolism**	**TSC2** (TSC Complex Subunit 2) [54]	Enhanced Protein Translation	Pim-2
	**4E-BP1** (Eukaryotic Translation Initiation Factor 4E Binding Protein 1) [55]	Enhanced Protein Translation; TSC2 target gene	Pim-2
	**PRAS40** (AKT1 Substrate 1) [56]	Increased mTOR activation of 4E-BP1 and p70S6K	Pim-1
	**eIF4B** (Eukaryotic Translation Initiation Factor 4B) [57]	Translation Initiation	Pim-1
	**LKB1** (Serine/Threonine Kinase 11) [58]	Cell Metabolism and Energy Production	Pim-1
**Apoptosis and Cellular Invasion**	**Bad** (BCL2 Associated Agonist of Cell Death) [59]	Anti-apoptotic release of Bcl-XL	Pim-1, −2, −3
	**Bim** (BCL2 Like 11) [60]	Anti-apoptotic	Pim-2
	**ASK1** (Apoptosis signaling kinase 1) [61]	Anti-Apoptotic to stress and inflammation	Pim-1
	**MDM2** (MDM2 Proto-Oncogene) [62, 63]	p53 degradation and transactivation	Pim-1 and 2
	**AR** (androgen receptor) [64]	Pro-migration and invasion [64, 65]	Pim-1
	**14–3-3ζ** [65]	AR co-activator	Pim-1
	**NKX3.1** (NK3 Homeobox 1) [66]	14–3-3ζ functional partner	Pim-1
**Lymphoid Signaling**	**NFATC1** (Nuclear Factor of Activated T Cells 1) [67]	T-cell signaling, bone formation, immunotherapy	Pim-1, −2, −3
	**SOCS-1/3** (Suppressor of Cytokine Signaling 1/3) [68, 69]	T-cell Regulation; JAK/STAT Signaling	Pim-1
	**FLT3** (Fms Related Receptor Tyrosine Kinase 3) [70]	STAT5a/b regulation and cytokine-sensitive signaling pathways	Pim-1
	**CXCR4** (C-X-C Motif Chemokine Receptor 4) receptor [71, 72]	Cell trafficking through ligands, such as CXCL12 (C-X-C Motif Chemokine Ligand 12)	Pim-1 and − 3
	**H19** [73]	lncRNA involved in stem cell signature	Pim-1
	**ABCB1 and ABCG2 (BCRP)** ATP Binding Cassette Subfamily G Member [31, 33]	Drug efflux; involved in drug resistance	Pim-1

**Fig. 4:**
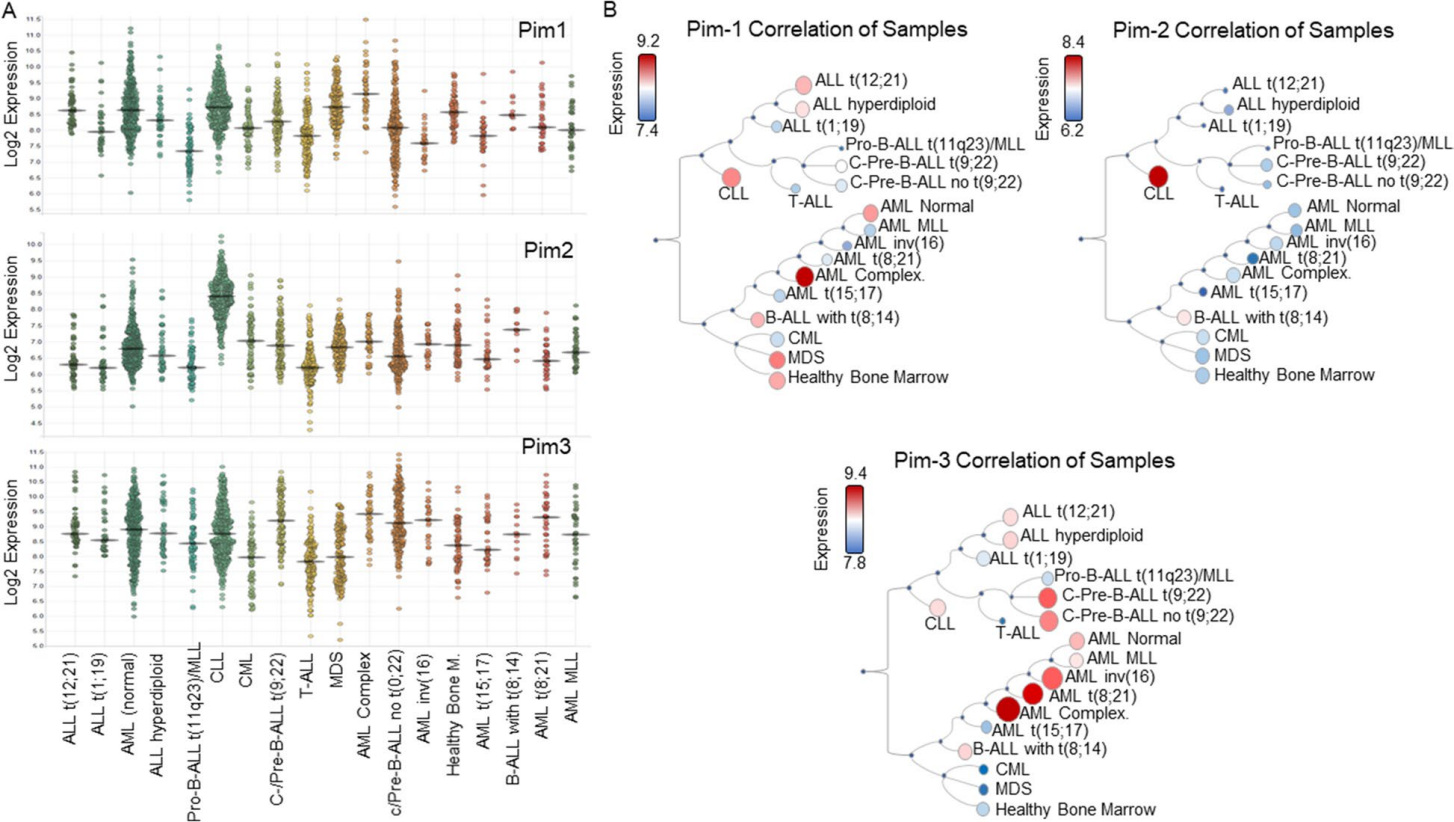
Microarray Expression of Pim kinases in AML, CML, ALL, CLL, and MDS. **A**-**B** Data are compiled from the BloodSpot Data base (https://servers.binf.ku.dk/bloodspot/) using expression data from the Microarray Innovations in Leukemia study (MILE) [11, 75]. Data are derived from four groups of leukemia: AML, CML, ALL, and CLL. The data are derived from 2095 patients, performed in various laboratories across the world. **A** Jitter strip chart demonstrating expression data of Pim-1, Pim-2, and Pim-3 across various subclasses of AML, CML, ALL, CLL, and MDS. **B** Hierarchical differentiation trees for the expression of Pim-1, − 2, and − 3 in AML, CML, ALL, CLL, and MDS. Pim kinase expression levels are indicated by the legend, whereby the size and color of the nodes indicates general expression patterns in various classes and subclasses of leukemia

As of 2020, the World Health Organization (WHO) reports the crude rate per 100,000 people worldwide of lymphoid cancers was 7.0, 6.1, 2.3, and 1.1 for non-Hodgkin’s lymphoma (NHL), leukemia, multiple myeloma (MM), and Hodgkin’s lymphoma (cHL), respectively. Novel therapeutics are needed to combat the rising incidence of lymphoid cancers. Gene expression profiling of hematological cancers, acute myeloid leukemia (AML), chronic myeloid leukemia (CML), acute lymphoblastic leukemia (ALL), chronic lymphocytic leukemia (CLL), and myelodysplastic syndromes (MDS), and their various subclasses, demonstrated high expression of Pim kinases across hematologic malignancies (Fig. 4A/B) [11, 12, 74, 75]. Pim-1 was highly expressed in complex AML, CLL, ALL, and MDS, while Pim-2 was especially prevalent in CML compared to other leukemias. Pim-3 showed higher expression in AML subclasses and c−/Pre-B-ALL. Given the strong role of Pim kinases in cancer progression, their over-expression in lymphoid cell lineage cancers, and their central role in several pro-tumorigenic pathways, Pim kinases represent an attractive therapeutic target. The following sections describe how Pim kinases are relevant in various lymphoid and myeloid cancers. These include, AML (acute myeloid leukemia), ALL (acute lymphocytic leukemia), and APL (acute promyelocytic leukemia); multiple myeloma (MM); lymphomas, that comprise non-Hodgkin’s lymphoma (NHL), Hodgkin’s lymphoma (cHL), and chronic lymphocytic leukemia (CLL); and chronic leukemias, that comprise, CLL, chronic myeloid leukemia (CML), adult T-cell leukemia (ATL), and myeloproliferative neoplasms (MPNs).

## Acute myeloid leukemia (AML)

**Fig. 5:**
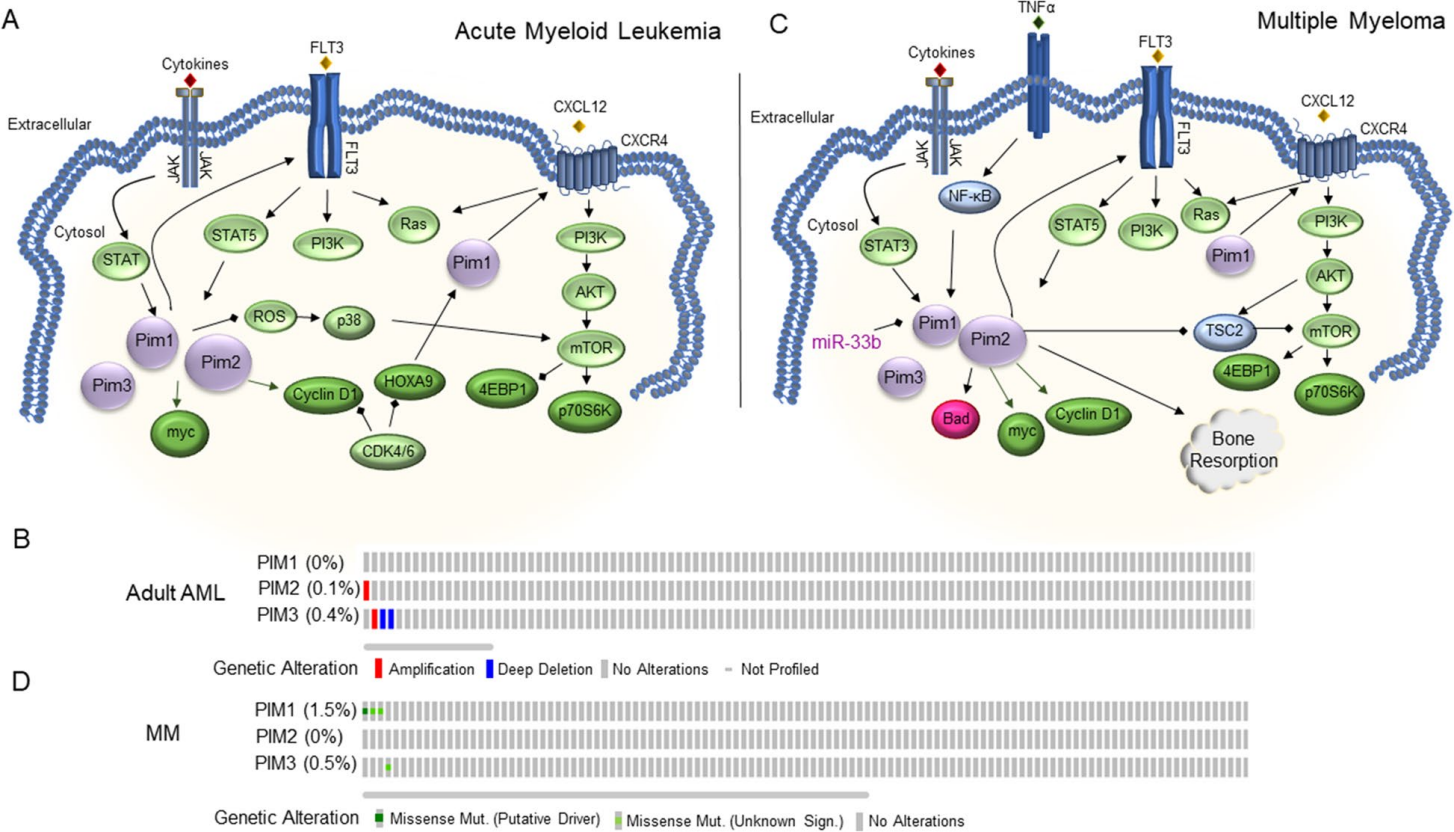
Pim Kinase Expression and Genetic Alterations in Acute Myeloid Leukemia (AML) and Multiple Myeloma (MM). **A** and **C** Model depicting Pim kinase regulation in AML (**A**) and MM (**C**). Pim kinases are depicted in purple, with relative expression depicted by the size of the node. **B** and **D** Oncoprints of genetic alterations in AML (**B**) and MM (**D**) for Pim kinases derived from the cBioPortal from Cancer Genomics [29]. For (**B**), the results published are from whole genome sequencing and/or whole exome sequencing generated by OHSU Beat AML Project and the TCGA AML Project [77, 78]. The Beat project encompassed 672 AML patients with 454 matched, normal samples, while the TCGA project included 200 adult de novo AML tumor/normal pairs. **D** Data are derived from the cBioPortal for Cancer Genomics. Data sourced to whole exosome sequencing of 203 paired MM and normal patient samples [85]

AML arises from the transformation of immature blast cells in the bone marrow through abnormal differentiation and genetic alterations, particularly in Fms-like tyrosine kinase 3 (FLT3), a driver mutation for AML. As much as 30% of AML patients have mutations in FLT3, with approximately 25% carrying constitutively activating internal tandem duplication mutations (FL3-ITD) [76]. FLT3 supports early hematopoietic and lymphoid cell proliferation. Expression of FLT3 or the presence of FLT3 mutations are linked to Pim kinases. While all three Pim kinases are over-expressed in AML patient samples, Pim-2 appears dominant (Fig. 5A). Genetic alterations in the Pim kinases, including mutations, are rare in AML patients. Whole exome sequencing verified a small percentage of adult AML patients harboring deep deletions and amplification of Pim-2 and Pim-3, but the clinical significance of this is unknown (Fig. 5B) [29, 77, 78]. Pim-1 expression regulates cellular homing and migration, an essential role that drives in vivo FLT3-ITD transformation of bone marrow cells [71]. This is in part due to Pim-1 increasing the expression and activation of CXCR4 (C-X-C Motif Chemokine Receptor 4) in AML blasts and hematopoietic cells in the bone marrow niche. CXCR4 drives mTOR signaling in AML. Even though Pim-2 is not sufficient to transform FLT3-ITD primary cells, Pim-2 has been shown to be necessary for the survival of immortalized, hematopoietic progenitor cells, that stably express FLT3-ITD [79]. In AML blasts derived from patient samples, the Pim-2 protein (and not Pim-1) was highly expressed compared to normal CD34+ cells [55]. Other studies have variable results and show high Pim-1 expression in AML patients, where Pim-1 and Pim-2 gene levels were higher in AML patient samples than those in complete remission. Furthermore, an association between high Pim-1 expression and higher risk groups and overall survival was reported [80, 81]. Pim-3 was also found to be over-expressed in the bone marrow derived samples from AML patients [82]. The difference in expression levels observed in different studies could be due to the lack of direct correlation between Pim-1 gene and protein levels, or to the fact that Pim-1 is not expressed in early, non-treated AML blasts, whereas Pim-1 is up-regulated in late stage, more aggressive cases. Indeed, microarray expression data demonstrates varying levels of Pim kinases in subclasses of AML [11, 75]. Pim-1 and Pim-3 expression were high in complex AML, whereas Pim-3 was also high in AML t(8;21) and AML inv.(16) compared to other AML subclasses (Fig. 4B). Additionally, Pim expression could correlate only with certain genetic mutations (ie. FLTD-ITD or FLTD-ITD), and therefore the percentage of AML patients in each data set harboring these mutations, which could explain varying levels of Pim kinases among studies. Indeed, wild-type FLT3 or expression of FLT3-ITD leads to increased Pim-1 expression [83]. STAT5 is downstream of FLT3 and JAK signaling, which are known to be potent inducers of Pim expression. Along with STAT5, the transcription factor, HOXA9 (homeobox A9), is up-regulated in AML and can also drive Pim-1 expression [84].

Acute promyelocytic leukemia (APL) is a type of AML characterized by an accumulation of immature granulocytes, promyelocytes, that harbor genetic translocations in the retinoic acid receptor alpha gene (RARα) that generates the PML/RARα gene, PRα. Mutated FLT3-ITD, along with PRα, is important in the pathogenesis and leukemogenesis of APL [86]. Pim-2 was found to cooperate with PRα to induce APL in mice, possibly by enhancing the likelihood of clonal events that lead to leukemia [87]. Accordingly, APL patient samples have high expression of Pim-2. Preclinical trials with Pim inhibitors have shown promising results. Treatment of newly diagnosed AML patients with K00135, an ATP-competitor of Pim kinases, leads to decreased cell viability in AML cells independent of FLT3-ITD, while the viability of normal, human cord blood cells was unchanged [88]. Treatment of the same patients with K00486, led to loss of Pim-1 regulated surface expression of CXCR4 without loss of cell viability [71]. AZD1208 and SGI-1776 also inhibit AML primary cells, xenograft AML tumors in animal models, and cell growth by down-regulating global RNA and protein synthesis [89–91]. These drugs inhibit Pim-mediated regulation of transcription and translation through c-Myc, 4E-BP1 (Eukaryotic Translation Initiation Factor 4E Binding Protein 1), and p70S6K (Ribosomal protein S6 kinase beta-1).

## Acute lymphoblastic leukemia (ALL)

**Fig. 6:**
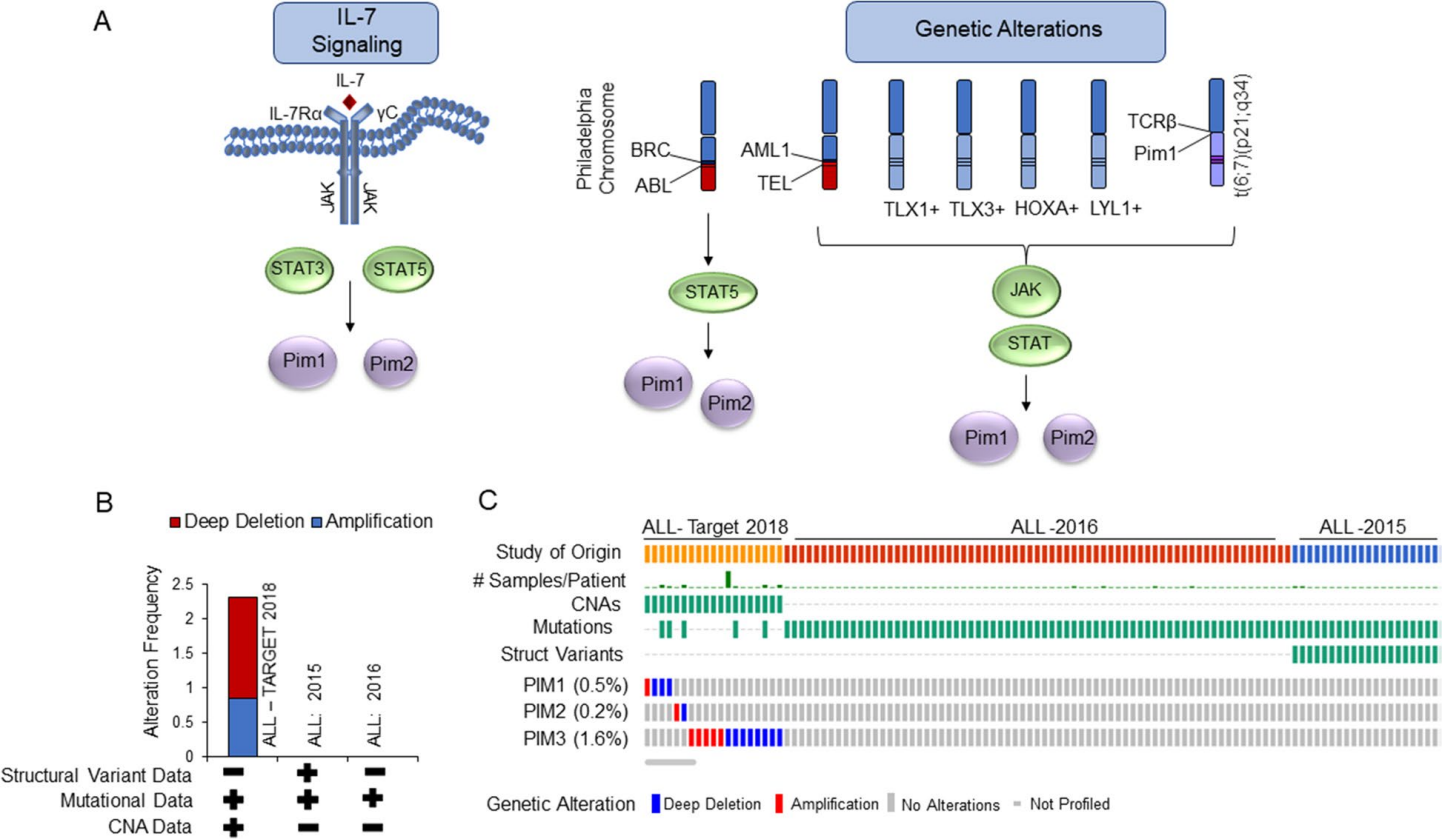
Cellular Signaling Pathways and Genetic Alterations Leading to Pim Kinase Expression in Acute Lymphoblastic Leukemia (ALL). **A** Model depicting the regulation of Pim kinase expression and activity in ALL. IL-7 signaling leads to enhanced Pim-1 and Pim-2 expression. Alternatively, common genetic alterations found in ALL patients leads to enhanced JAK/STAT activity and up-regulation of Pim kinases. **B**-**C** Cancer type summary (**B**) and OncoPrint (**C**) of genetic alterations from 3 cancer genomic studies on ALL. Data are derived from the cBioPortal for Cancer Genomics [29, 30]. The results published here (ALL, Target-2018) are in whole or part based upon data generated by the Therapeutically Applicable Research to Generate Effective Treatments (TARGET) https://ocg.cancer.gov/programs/target initiative, phs000464 (Acute Lymphoblastic Leukemia (ALL) Expansion Phase 2). The data used for this analysis are available at https://portal.gdc.cancer.gov/projects. Target-2018 are derived from pediatric ALL. ALL-2016 are derived from 69, primarily children and young adult, B-progenitor ALL patient samples [103]. ALL-2015 are derived from 85 ALL infants with MLL rearrangements [104]

The most common type of ALL is precursor B-lymphoblastic leukemia (B-ALL) which accounts for 88% of childhood ALL, and 75% of adult ALL. A database analysis demonstrated high expression of Pim kinases in different subgroups of ALL patients, where Pim-1 expression was the highest in ALL patient samples (*n* = 350) when compared to AML, MM, and DLBCL samples [92]. Pim-3 is also elevated in ALL compared to other leukemias. Bone marrow mononuclear cells from B-ALL patients over-express Pim-1 and Pim2, whereas Pim-3 expression was unchanged compared to normal cells [93]. Pim-1 is also highly expressed in pediatric T-ALL patient samples, especially the early T-cell precursor, ETP-ALL subtype, suggesting Pim-1 is a driver of early T-ALL growth compared to more mature T-ALL disease [94]. Commutatively, these observations demonstrate that Pim kinases are active in ALL; and evidence strongly links Pim activation to genetic translocation or mutation/activation of the JAK/STAT and/or interleukin-7 (IL-7Rα) pathways (Fig. 6A). Most primary samples from T-ALL express IL-7Rα and respond to IL-7 [95, 96]. Pim-1 is activated downstream of the IL-7Rα, through STAT5, either through mutation or through IL-7 stimulation [97, 98]. A large percentage of ALL cases harbor genetic abnormalities in tyrosine kinases. The BCR/ABL-Ph + translocation (breakpoint cluster region/Proto-oncogene tyrosine-protein kinase ABL1), involving the Philadelphia chromosome (Ph+) translocation is the most common genetic translocation found in adult ALL cases, occurring in approximately 25% of cases. Though Pim-1 expression is not required for BCR/ABL-mediated transformation, it does benefit tumor cell survival. While the BCR/ABL is the most common genetic translocation in adult ALL, the TEL/AML1 (Transcription Factor ETV6/RUNX Family Transcription Factor 1) fusion is the most common in childhood ALL, occurring in approximately 22% of cases. Though rare, the BCR/ABL-Ph + translocation has also been reported in T-ALL. In addition to TEL/AM1, chromosome aberrations in chromosome 6 are found in B-ALL patients. In a small case study of TEL/AML1+ ALL childhood samples, 86% harbored additional chromosomal aberrations, including chromosome 6, where the Pim-1 gene (6p21–23) is located [99–101]. Though not confirmed it does suggest that genetic aberrations in the Pim-1 gene could contribute to leukemogenesis in B-ALL. Further supporting this, cells transformed by oncogenic tyrosine kinases (TEL/JAK2, TEL/TRKC (TEL/tyrosine kinase receptor C), TEL/ABL, BCR/ABL, FLT3-ITD, and H4/PDGFβR (D10S170/Platelet derived growth factor receptor beta)) all display elevated levels of Pim-1 and Pim-2 expression [102]. In addition, large genetic screens of ALL patients found a small percentage of genetic alterations in mostly pediatric ALL samples (Fig. 6B) [29, 30, 103, 104]. These consisted mostly of deep deletions in the Pim-3 gene, with gene amplifications in all 3 Pim kinases (Fig. 6C). The significance of these alterations, and whether they lead to enhanced Pim kinase activity, is currently unknown. T-cell acute lymphoblastic leukemia (T-ALL) comprises 12% of ALL in children and 25% in adults. Pim-1 is one of the critically deregulated oncogenes in T-ALL. Similar to B-ALL, genetic aberrations in Pim-1 gene locus have been found in T-ALL and T-LBL patients, t(6;7)(p21;q34), producing, TCRβ-PIM1 (T-cell receptor beta locus/PIM1), whereby the TCRβ enhancer was found juxtaposed to the 5’UTR (untranslated region) of the Pim-1 gene [105–108]. Although this genetic translocation is rare in T-ALL patients, TCRβ-PIM1+ T-ALL patients and T-ALL subgroups (HOXA, TLX, and LYL1+ (Lymphoblastic leukemia derived sequence 1)) that have JAK/STAT activating abnormalities (JAK1, JAK2, JAK3, IL7R, STAT5A/B, PTPN2 (protein tyrosine phosphatase non-receptor type 2), and/or NUP214-ABL1 (Nucleoporin Nup214-ABL1)) all lead to very high levels of Pim-1 gene expression [105, 108].

## Multiple myeloma (MM)

MM is characterized by over-proliferating plasma cells in the bone marrow. Pim-1, − 2, and − 3 are expressed in MM cell lines and in primary MM patients [109] (Fig. 5C). However, Pim-3 expression is not strongly associated with the occurrence of MM cells. Pim-2 is highly expressed in MM cells and Pim-2 knock-down studies demonstrate that Pim-2 is required for MM cell proliferation [54]. Pim-2 expression is up-regulated in MM cells through IL-6/JAK2/STAT3 and TNFα/NF-κB mediated pathways and inhibitors to either STAT3 (cucurbitacin I) or IKKα/β (I-Kappa-B Kinase)(parthenolide) decrease Pim-2 expression [110]. Mutations, including hot spot, putative driver mutations in Pim-1, have been found in MM patient samples (Fig. 5D) [29, 30, 85]. Pim-2 may also play an important role in osteolytic bone lesions often seen in MM patients. In fact, high expression of Pim-2 has been detected in osteoclasts and bone marrow stromal cells in the MM microenvironment [110]. This was associated with worsened osteoclastic functions and bone lesions in MM patients. In agreement with these observations, use of Pim kinase inhibitors provide some protective effect on bone disease in MM patients [109, 111, 112]. This was found to be at least partially due to Pim-2-mediated inhibition of osteoblastogenesis and Pim regulation of NFATC1 (Nuclear factor of activated T-cells 1) and cathepsin K.

## Non-Hodgkin’s lymphoma (NHL), chronic lymphocytic leukemia (CLL), and Hodgkin’s lymphoma (cHL)

**Fig. 7:**
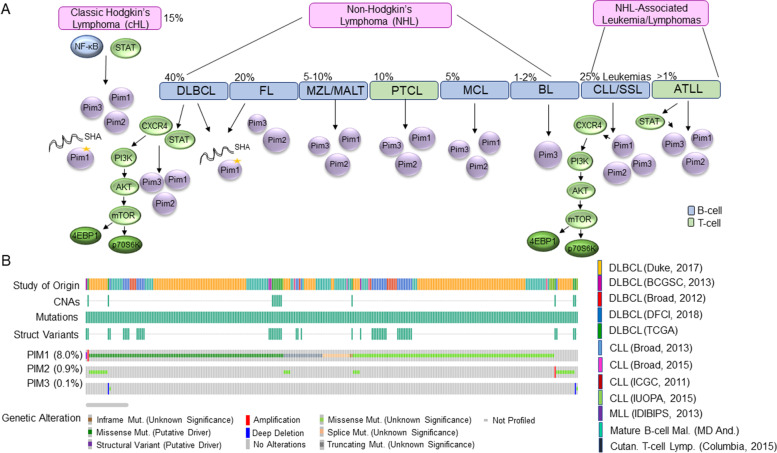
Cellular Signaling and Genetic Alterations in Hodgkin’s and non-Hodgkin’s leukemia/lymphoma Leading to Enhanced Pim Kinase Activity. **A** Model depicting the regulation of Pim kinase expression and activity in various Hodgkin’s and non-Hodgkin’s lymphomas/leukemias. These include cHL (Classic Hodgkin’s Lymphoma), DLBCL (Diffuse Large B-cell Lymphoma), Follicular Lymphoma (FL), Marginal zone lymphoma (MZL), Mucosa-associated Lymphoid Tissue (MALT) lymphoma, Peripheral T-cell lymphoma (PTCL), Mantle Cell Lymphoma (MCL), Burkitt lymphoma (BL), Chronic lymphocytic leukemia (CLL), Small lymphocytic lymphoma (SLL), and Adult T-cell leukemia/lymphoma (ATLL). **B** OncoPrint of genetic alterations from 12 cancer genomic studies on various lymphomas and leukemias. Data was collected from cBioPortal for Cancer Genomics [29, 30]. These include whole genome sequencing from 4 chronic lymphocytic leukemia (CLL) studies: CLL from Broad-2013 [114], CLL tumors and normal samples from Broad-2015 [115], CLL, Monoclonal B-cell lymphocytosis (MBL), and 24 SLL from ICGC-11 [92], and CLL from IUOPA-2015 [93]. Whole genome sequencing from 5 diffuse large B-cell lymphoma (DLBCL) studies, including normal samples: DLBCL from Duke-17 [97], DLBCL from BCGSC-13 [95], DLBCL from Broad-12 [96], DLBCL from DFCI-18 [94], and DLBCL from TCGA [98]. Whole genome sequencing from cutaneous T-cell lymphomas from 25 Sezary syndrome and cutaneous T-cell lymphomas (CTCL) from Columbia-15 [99], mantle cell lymphoma (MCL) from IDIBIPS-13 [100], and mature B-cell malignancies from MD Anderson [101]

Non-Hodgkin’s lymphoma (NHL) encompasses a large array of leukemia/lymphomas, with over 60 subtypes recognized by the WHO. Nearly 85% of all NHLs are of B-cell origin. Aggressive NHLs include, diffuse large B-cell lymphomas (DLBCL) and its subtypes, Burkitt’s lymphoma (BL), peripheral T-cell lymphoma (PTCL), Mantle cell lymphoma (MCL), and transformed mucosa-associated lymphoid tissue (MALT) lymphoma and more indolent NHLs, including, chronic lymphocytic leukemia (CLL)/small lymphocytic lymphoma (SLL), follicular lymphoma (FL), cutaneous T-cell lymphoma (CTL), and nodal marginal zone lymphoma (NMZL). While an examination of Pim kinase expression, activity, and Pim kinase inhibitor therapy for all 60 NHL subtypes is beyond the scope of this review, there are key takeaways for NHLs. Most NHLs over-express Pim-1/Pim-2 – as an examination of patient samples from indolent and aggressive NHLs (MCL, DLBLC, FL, MZL-MALT, CLL, and NMZL) showed marked over-expression of Pim-2, and to a lesser degree, Pim-1, with Pim-3 levels similar to normal lymph node and tonsil tissues [113]. There are notable exceptions with BL patient samples displaying high levels of Pim-3 expression; and MCL expressing more Pim-1, while Pim-2 was only expressed in conjunction with Pim-1 [24, 63]. In addition, most NHLs are susceptible to Pim kinase inhibition either in mono or dual therapy; and the Pim-1 gene is susceptible to somatic hypermutation activity in some NHL subtypes (Fig. 7A).

The most common subtype of NHL, DLBCL, compromises 30–40% of all NHL. DLBCL can occur from FLs and CLLs; and originates in germinal center B-cells. DLBCL patient samples express high levels of all Pim kinases, that correlate with active STAT3/STAT5/CXCR4. Survival and proliferation of these tumor cells rely upon activation of Pim as demonstrated by use of Pim inhibitors [113, 116]. There are two main molecular subtypes of DLBCL classified by their different source cells, stage of B-cell differentiation, and clinical outcomes: germinal center (GC-DLBCL) and activated B-cell-like DLBCL (ABC-DLBCL). Overall survival is lower in DLBCL patients expressing Pim-2, which appears to be restricted to the ABC subtype [113]. Pim-2 expression was found to be higher in the ABC-DLBCL subtype than the GC subtype; whereby Pim inhibition with ETP-39010 or Pim2 knock-down decreased BAD (Bcl2 associated antagonist of cell death), AKT, and 4E-BP1. There are numerous special subgroups of DLBCL. Primary mediastinal large B-cell lymphoma (PMLBCL) is a subgroup of DLBCL, while both nodular lymphocyte-predominant HL (NLPHL) and classic Hodgkin’s lymphoma (cHL) also arise from different differentiation stages in germinal center B-cells. A unique feature of these lymphomas, DLBCL, PMLBCL, NLPHL, and cHL, is the frequency of somatic hypermutation activity (SHA) that leads to genome instability. SHA is a common process of mutation of the immunoglobulin variable (IGV) region of B-cells undergoing antigen primed maturation. In contrast to most leukemias, the Pim kinases are subject to high rate of genetic alterations in lymphomas. Whole genome sequencing finds that almost 8% of DLBCLs, CLL, MLL, mature B-cell lymphomas, and cutaneous T-cell lymphomas carry Pim-1 genetic alterations (Fig. 7B) [29, 92–101, 114, 115]. These encompass, putative driver missense mutations, and amplifications, splice, missense, in frame, and truncating mutations of unknown significance. Pim-1 is one of four proto-oncogenes involved in DLBCL, PMLBCL, and FL SHA [102, 105, 106]. The 5’UTR of Pim-1, along with Pax-5 (Paired Box 5), RhoH/TFF (Ras homolog family member H/Trefoil Factor), and c-Myc, undergoes SHA with mutations occurring within coding exons leading to changes in amino acids and possibly Pim-1 activity. These genes, including Pim-1, are also highly susceptible to chromosomal translocations and double-strand DNA breaks [107]. SHA of the Pim-1 gene has also been shown for AIDS-related non-Hodgkin lymphomas (AIDS-NHLs) and HCV-positive B-cell NHL patients [108, 117]. In DLBCL, and particularly the subtype ABC-DLBCL, Pim-1 is one of the highest mutated genes [118]. The missense and frameshift mutations found in DLBCL due to SHA preserve Pim-1 functionality and tumor cells remain sensitive to PIM447 inhibition. These events were found to be only slightly correlated with negative disease prognosis. Even then, these phenotypes were mostly believed to arise from aberrant AID activity itself, and not Pim mutation [118]. In another subtype of NHL, primary central nervous system lymphoma (PCNSL), Pim-1 is one of the most highly mutated genes and over 77% of PCNSL harbored Pim1 mutations [119]. Pim-1 expression was high in PCNSL patients and correlated with MYD88 (Myeloid Differentiation Primary Response Protein MyD88) expression. High expression of either of these proteins led to a more unfavorable prognosis and lower overall survival. Therefore, Pim-1, along with other proto-oncogenes, may have a substantial role in the development of lymphomas and presents as an interesting therapeutic target. MCL is an NHL in which patients are characterized as having high levels of cyclin D1, with patients that go through patterns of continuous relapse following chemotherapy. MCL subtypes have shown especially high levels of Pim-1 and Pim-2 [120]. Preclinical use of the Pim inhibitor SGI-1776 resulted in decreased phosphorylation of translational targets (4E-BP1) associated with a lower expression of cyclin D1 and survival factor MCL1 (BCL2 family apoptosis regulator) leading to apoptosis of tumor cells [90].

T-cell lymphomas make up for less than 15% of all NHLs. These include lymphomas of the mature T-cells located in the periphery, such as primary peripheral T-cell lymphoma (PTCL) and adult T-cell leukemia/lymphoma (ATLL). In PTCL patient samples, Pim-1 and Pim-2 expression is elevated and correlates with STAT, NF-κB, and IL-2 (interleukin-2) compared to normal lymph nodes [121]. PTCL cells treated with the pan-Pim inhibitor, ETP-39010, lost viability due to apoptosis induction and an impaired DNA damage response. ATLL (adult T-cell leukemia/lymphoma) is a chronic leukemia/lymphoma deriving from mature T-cells infected with the human T-cell leukemia virus type-1 (HTLV-I). Leukemic patient cells from ATLL patients exhibit high expression of all Pim kinases with over-expression of Pim-2 and Pim-3 when compared to normal PBMCs (peripheral blood mononuclear cells) [122]. All three Pim kinases are regulated by the JAK/STAT pathway, particularly STAT3 and STAT5. Compared to normal PBMCs, ATLL cells are sensitive to Pim kinase inhibition (Smi-4a and AZD1208) and ex vivo ATLL tumor burden is decreased in mice treated with AZD1208 [122]. Since ATLL is caused by the HTLV-I virus, a role for Pim-1, − 2, and − 3 in inhibiting viral transactivation has also been seen, which may allow immune escape and sustainment of ATLL cells [123].

CLL is the most common chronic leukemia in adults. While CLL is not by definition an NHL, CLL patients can develop NHL through Richter’s syndrome. Studies have confirmed over-expression of Pim-1 and Pim-2 in CLL patients compared to normal lymphocytes [124, 125]. Pim-1/− 2 are especially elevated in B-CLL patients and correlated with worse prognosis in B-CLL (elevated in Binet stage C) and B-CLL subtype, while Pim-1/2 expression was lower in patients achieving complete remission [126–129]. Pim-3 did not correlate with any clinical characteristic. Like AML, CXCR4 phosphorylation, a hallmark of CLL cells, correlates with Pim-1 kinase expression [124]. CLL cells respond favorably to Pim kinase inhibitors– whereby K00135 and K00486 (two inhibitors which preferentially target Pim-1/− 2) and the pan-Pim inhibitor, A47, decreased CLL cell proliferation and apoptosis [124]. In addition, treatment with SGI-1776 inhibited ex vivo primary CLL patient samples by way of Mcl-1 mediated apoptosis and decreased RNA transcription [125]. Use of the Pim inhibitor, SEL24/MEN1703 (SEL24-B489), in ex vivo primary CLL patient samples led to similar effects that were not seen in normal B-cells [129]. Knock-down of individual Pim kinases in CLL cells demonstrated that Pim-1 regulates CXCR4 surface expression, while Pim-2 and Pim-3 are more important for CLL survival [124]. Therefore, the use of Pim kinase inhibitors leads to a loss in CLL chemotaxis due to less phosphorylation and expression of CXCR4 on the cells surface and loss of CXCR4 activation of mTOR [129].

The most common B-cell lymphoma outside of NHLs, are classic Hodgkin’s lymphoma (cHL). cHL is characterized by Reed-Sternberg cells, enlarged B-cells that express few B-cell specific genes. Aside from SHA of the Pim-1 gene that is also seen in DLBCL, cHL patient samples also express Pim kinases through normal transcriptional means. Patient samples from cHL have high expression of Pim-1,-2, and − 3 driven by NF-κB and STAT pathways and are sensitive to Pim kinase inhibition with the Pim/FLT3 inhibitor, SEL24/MEN1703 (SEL24-B489), or the dual Pim kinase combined with histone deacetylase inhibitor (suberoylanilide hydroxamic acid (SAHA) [130, 131]. Pim inhibition in cHL modulates expression of immunoregulatory molecules (PD-L1/2 and Gal-1) creating an immunosuppressive tumor microenvironment.

## Myeloproliferative neoplasms (MPN)

**Fig. 8:**
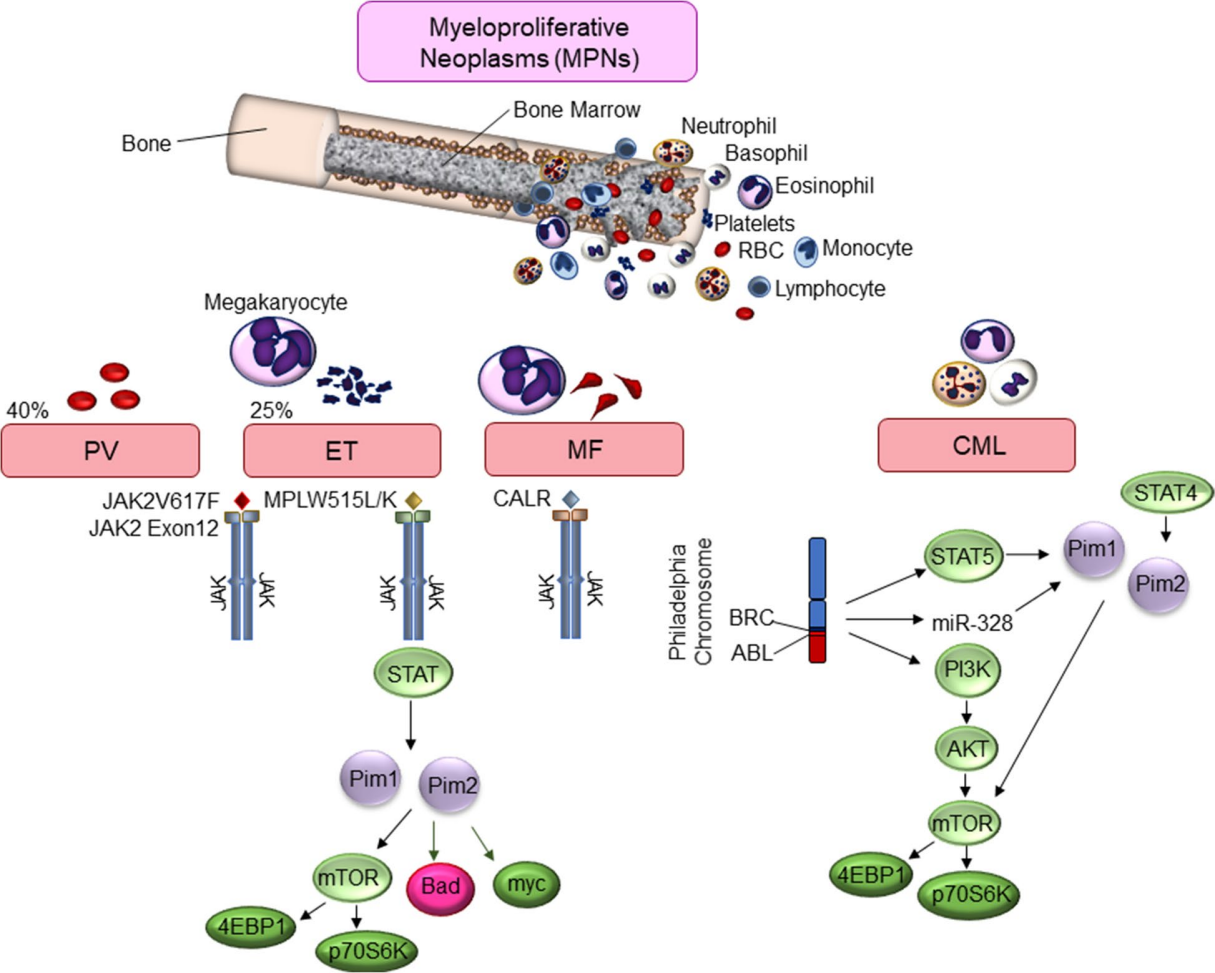
Cellular Mechanisms for Increased Pim Kinase Activity in Myeloproliferative Neoplasms. Model depicting the regulation of Pim kinase expression in various myeloproliferative neoplasms (MPNs). These include Polycythemia Vera (PV) blood cancer, Essential Thrombocythemia (ET) blood cancer, Myelofibrosis (MF) blood cancer, and Chronic Myelogenous Leukemia (CML). These cancers arise from mature hematopoietic cells in the lymph nodes. Pim kinases are activated through various mechanism, including genetic alterations JAK/STAT pathway and BRC/ABL mutations leading to downstream activation of Pim kinases

MPNs and CML comprise a class of chronic leukemias that arise from mature hematopoietic cells in the lymph nodes. MPNs are classified into Philadelphia chromosome-positive (Ph+) or -negative (Ph-) categories. Most MPNs are considered classical MPNs, without Ph- translocations and are divided into three main types, polycythemia vera (PV), myelofibrosis (MF), and essential thrombocythemia (ET). MPNs occur when bone marrow stem cells grow abnormally and over-produce leukocytes, such as red blood cells (PV), platelets (ET), granulocytes (CML), or neutrophils/eosinophils. MF is the deadliest MPN whereby scar tissue develops in the bone marrow, causing a disruption in normal leukocyte production, with enhanced megakaryocytes. MPNs are characterized not only by their cytology, but also the high level of JAK2 mutations. 95% of PVs and 50–60% of ETs and MFs harbor JAK2 mutations [132]. In addition, mutations in calreticulin (CALR) and MPL (thrombopoietin receptor), particularly the MPLW515L/K mutation, which signals through JAK2, are frequently seen. Pim-1/− 2 expression is dependent upon JAK2 signaling in MPN (Fig. 8) [133]. In PV patient samples carrying the constitutively active JAK2 mutation, V617F, Pim-1 is over-expressed due to an active JAK/STAT pathway compared to non-V617F PV patients samples [134]. Pim-1 is highly over-expressed in granulocytes, PBMCs, and bone marrow from MF patient samples [135]. Pim-1 cooperates with JAK or MPL mutants in the development of MF. Loss of Pim-1 gene expression or treatment with Pim inhibitors (TP-3654) in various in vivo MF models, involving JAK V617F or MPL515L, led to loss of MF development and decreased bone fibrosis. Furthermore, in vitro cell data demonstrated that V617F transformation in erythroleukemia cells was dependent upon downstream JAK signaling components, c-Myc, Pim-1, and Pim-2 [136].

CML results from hematopoietic stem cells of myeloid origin. As much as 90% of CML patients carry the Ph + translocation, which results in a shortened chromosome 22. Like ALL, this results in the oncogene tyrosine kinase gene, BCR-ABL. Pim-1 is up-regulated downstream of BCR-ABL through STAT5 induction and miR-328 mediated regulation of the Pim-1 3’UTR [137]. In CML patients Pim-1 is essential for BCR-ABL mediated in vivo transformation and leukemogenesis [138]; and simultaneous knockdown of Pim-1 and Pim-2 in cells transformed by BCR-ABL eliminated growth factor dependent growth [139]. These results suggest Pim kinase inhibition could also be an effective treatment against CML.

## Targeting Pim kinases in leukemia and lymphoma

Pim kinases have a unique ATP binding mode, which unlike other kinases has a single hydrogen bond donor in the hinge region [140]. This structural difference has allowed the development of specific and selective inhibitors that target Pim kinases. First and second-class generations of Pim kinase inhibitors have been developed over the past decade (Table 2). Early Pim inhibitors, or 1st generation inhibitors, targeted solely Pim-1, without much efficacy against other isoforms. However, given the redundancy in some Pim kinase substrates it was soon realized that an inhibitor that blocked all three kinases was needed to block Pim signaling. Subsequently, inhibitors were developed that inhibited all three Pim kinases (pan-Pim kinase inhibitors, or 2nd class Pim inhibitors). These include most of the Pim kinase inhibitors used in clinical trials. SuperGen’s, SGI-1776, was the first, and only 1st generation Pim inhibitor to be used in human clinical trials. While showing limited efficacy in solid tumors and lymphomas, SGI-1776 caused adverse cardiac QTc prolongation events, leading SGI-1776 to be discontinued. The 2nd generation Pim inhibitor AZD1208, developed by AstraZeneca, entered Phase I human trials in 2012. Dose escalation studies were originally performed in advanced solid tumors and hematological malignancies. However, clinical results did not meet primary endpoints; and AZD1208 was associated with adverse events suggesting it would not be clinically useful [141]. More recently, two new 2nd class inhibitors, PIM447 (LGH447) and Uzansertib (INCB053914) have been developed. PIM447, developed by Novartis, shows greater pan-Pim inhibition than either AZD1208 or SGI-1776, enhancing the block against Pim-2 [142]. PIM447 was also effective at significantly lower doses and with low to moderate clearance. As of this review, PIM447 has been used in at least 5 clinical trials, with results from one limited study demonstrating positive results (discussed below for MM). Incyte Corporation developed Uzansertib as a 2nd class generation therapy with potent toxicity against all three Pim isoforms [143]. Uzansertib has been part of 3 Phase 1 clinical trials. While one study was terminated due to a lack of funding, another found possible efficacy as a monotherapy in advanced hematological malignancies (discussed below for AML) [144].

**Table 2:** Current Pim inhibitors used in vitro, ex vivo*,* and in current or past clinical trials

	Drug Target	Development	Clinical Trials	Treatment Group	Therapy Type
**A47**	pan-PIM	Preclinical	–	–	–
**Abemaciclib (Verzenio)**	Pim1-CDK4/6	Phase 1	NCT03905889: Active (NR)	Metastatic Renal Cell Carcinoma	Dual therapy with Abemaciclib and Sunitinib
**AZD1208**	pan-PIM	Phase 1 Phase 1	NCT01588548: Completed NCT01489722: Terminated	Adv Solid Tumors/Malignant Lymphoma Relapsed/Refractory AML	Monotherapy Monotherapy
**AZD1897**	pan-PIM	Preclinical	–	–	–
**ETH-155008**	Pim3-CDK4/6 (FLT3)	Phase 1	NCT04840784: Recruiting	R/R: B-NHL, CLL/SLL and AML	Monotherapy
**ETP-39010** **ETP-45299** **ETP-47551**	pan-PIM	Preclinical	–	–	–
**INCB053914 (Uzansertib)**	pan-PIM	Phase 1 Phase 1/2 Phase 1	NCT03688152: Completed NCT02587598: Ter/BD NCT04355039: WD/LF	Relapsed/Refractory DLBCL Adv Solid Tumors Relapse/Refractory MM	Dual therapy with INCB050465 Monotherapy and with cytarabine, azacitidine, and ruxolitinib Triple agent therapy with pomalidomide with dexamethasone
**JP11646**	Pim2	Preclinical	–	–	–
**K00135**	Pim1/Pim2	Preclinical	–	–	–
**K00486**	Pim1/Pim2	Preclinical	–	–	–
**LGB321**	pan-PIM	Preclinical	–	–	–
**PIM447 (LGH447)**	pan-PIM	Phase 1 Phase 1 Phase 1 Phase 1 Phase 1	NCT02370706: Completed NCT01456689: Completed NCT02078609: Completed NCT02144038: Completed NCT02160951: Completed	Myelofibrosis Relapsed/Refractory MM AML or High Risk MDS Relapsed/Refractory MM Relapsed/Refractory MM (Japan)	Triple agent therapy with Ruxolitinib and LEE011 Monotherapy Monotherapy or Dual therapy with midostaurin (AML only) Dual agent therapy with BYL719 Monotherapy
**SEL24/MEN1703 (SEL24-B489)**	PIM/FLT3	Phase 1/2	NCT03008187: Recruiting	AML	Dual Pim/FLT3 therapy
**SGI-1776**	Pim1	Phase 1 Phase1	NCT01239108: Withdrawn NCT00848601: Terminated	Relapsed/Refractory Leukemias Refractory Prostate and Refractory NHLs	Monotherapy Monotherapy
**Smi-4a** **Smi-16a**	PIm1 Pim1/Pim2	Preclinical	–	–	–
**TP-3654**	Pan-PIM	Phase 1/2	NCT04176198: Recruiting	Intermediate/high-risk primary or secondary MF	Monotherapy

At least three, Phase 1 clinical trials with Uzansertib have been conducted in advanced hematological malignancies, which included AML. Early data suggests Uzansertib was favorably tolerated, inhibited Pim activity, and had few adverse effects (that could be treated with reduced Uzansertib or additional therapy) [144]. This trial included advanced AML, high-risk MDS, MPN, MM, and lymphomas. The most common adverse effects were elevated alanine aminotransferase (ALT) and aspartate aminotransferase (AST) levels, which could be suggestive of liver or heart issues. Phase 1 clinical trials on AML patients using AZD1208 or PIM447 have also been performed. Monotherapy with AZD1208 was unable to clear disease and had adverse events, particularly related to gastrointestinal effects of nausea and diarrhea [141]. Febrile neutropenia and rash were also noted. While monotherapy with Pim inhibitors showed promise for treating AML, (particularly Uzansertib and PIM447) intrinsic resistance to Pim inhibition has made dual-therapeutic options a more attractive option. Addition of FLT3-ID inhibitors to AZD1208 is effective in enhancing apoptosis in primary AML blasts due to the positive feedback loop that exists between Pim-1 and FLT3 [70, 145]. Based on the fact that Pim loss leads to increases in ROS/p38/AKT/mTOR signaling (Fig. 3 and Table 3); dual targeting with a p38 inhibitor (SCIO-469), AKT inhibitors (MK2206 and AZD5363), mTOR inhibitors (AZD8055 and AZD2014), or PI3K inhibitors (GDC-0941) have all shown beneficial effects [146–150]. Dual chemotherapy of primary FLT3-ITD AML cells with topoisomerase inhibitors (daunorubicin, etoposide, and mitoxantrone) and AZD1208 also sensitizes cells because of accumulation of reactive oxygen species (ROS) and DNA double strand breaks [151, 152]. EC-70124, a multi-kinase drug targeting FLT3, JAK, SYK, and Pim kinases was also efficient at targeting AML cells [153]. Enhanced efficacy with dual pan-Pim kinase inhibitors and CDK4/6 (PD 0332991), JAK/STAT5 (ruxolitinib) inhibitors, or CXCR4 antagonists (BL-8040 and Plerixafor) that are in clinical trials, may also prove beneficial in treating refractory AML [154–159]. In fact, a Phase I human clinical trial has recently begun recruiting for the use of ETH-155008, a triple inhibitor to Pim-3, FLT3, and CDK4/6 in the treatment of relapsed/refractory AML, CLL/SLL, and B-cell NHL. ETH-155008 has shown promise in pre-clinical trials with AML cell lines and in vivo AML mouse models [160]. SEL24/MEN1703 is another dual-therapy drug showing promise in AML. SEL24/MEN1703 is a PIM/FLT3 inhibitor currently recruiting for Phase I/II clinical trials. Early results indicate SEL24/MEN1703 may be effective as a single use agent in AML. SEL24/MEN1703 had a manageable safety profile and complete remission was seen in several patients [161].

**Table 3:** Pim kinase inhibitors used in preclinical and clinical (bold) trials for AML

Acute Myeloid Leukemia	Target(s)	Type	Clinical Trials	
**AZD1208**	**pan-Pim**	**Single**	**AML**	**NCT01489722**
AZD1208 + Daunorubicin (etoposide, and mitoxantrone)	pan-Pim + Topoisomerase 2	Dual-therapy	Preclinical	–
AZD1208 + SCIO-469	pan-Pim + p38	Dual-therapy	Preclinical	–
AZD1208 + MK2206 (AZD5363)	pan-Pim + AKT	Dual-therapy	Preclinical	–
AZD1208 + AZD2014 (AZD8055)	pan-Pim + mTOR	Dual-therapy	Preclinical	–
AZD1897	pan-Pim	Single	Preclinical	–
EC-70124	pan-kinase (Pim, FLT3, JAK, SYK)	Single	Preclinical	–
**ETH-155008**	**Pim3** **FLT3** **CDK4/6**	**Triple inhibitor (FLT3, Pim-3 and CDK4/6) Monotherapy**	**AML**	**NCT04840784**
ETP-45299 + GDC-0941	Pim1 + PI3K	Dual-therapy	Preclinical	–
K00135	Pim1/Pim2	Single	Preclinical	–
K00486	Pim1/Pim2	Single	Preclinical	–
**PIM447**	**pan-Pim**	**Single**	**AML**	**NCT02078609**
**SEL24/MEN1703**	**PIM** **FLT3**	**Double inhibitor (FLT3, Pim) Monotherapy**	**AML**	**NCT03008187**
SGI-1776	pan-Pim	Single	Yes	–
**Uzansertib**	**Pan-Pim**	**Single**	**AML**	**NCT02587598**

To date, there are currently no human clinical trials ongoing or recruiting for treatment of ALL with Pim inhibitors. However, extensive pre-clinical testing suggests this could be an interesting option to reduce ALL burden (Table 4). Inhibition of Pim kinases in Ph + ALL, including relapsed and refractory Ph + ALL, leads to robust reductions in colony formation, which are further enhanced with the addition of the Bcl-2 antagonist, sabutoclax [162]. This combination is also effective against leukemia burden in mice ejected with Ph + ALL cells. Treatment with Pim inhibitors (AZD1208 or TP-3654) in T-ALL or T-LBL patient derived xenograph mice models carrying the TCRβ-PIM1+ translocation or high Pim-1 expression levels caused tumor growth to be substantially inhibited [163]. Significant survival was further seen in these mouse models when dexamethasone, a glucocorticoid used in current ALL treatment, was given in parallel. T-ALL cells are also sensitive to Pim inhibitors (AZD1208 and LGB321) to varying degrees depending upon the level of Pim-1, JAK/STAT, IL-7, and/or the sensitivity to Notch gamma secretase inhibition [164, 165]. Combined Pim and tyrosine kinase inhibitor (ponatinib and dasatinib) therapy led to synergistic effects; with decreased tumor burden and prolonged survival in T-ALL engrafted mice [165]. Pim inhibitor therapy (Smi-4a) could also synergize with mitogen-activated protein kinase kinase 1/2 (MEK1/2) inhibitor (U0126) in reducing tumor burden in T-ALL and B-ALL engrafted mouse tumors [166, 167]. Interestingly, glucocorticoids that are given during the early, induction phase of T-ALL and T-LBL treatment, can induce IL-7Rα activity. This suggests that tumor cells surviving initial treatment may over-express Pim-1, given that IL-7 activity leads to JAK/STAT activation. Indeed, mouse xenograph models of T-ALL/T-LBL treated with a standard chemotherapy regimen (vincristine, dexamethasone and L-asparaginase) showed strong up-regulation of Pim-1 in residual leukemic blasts [168]. Increased survival was noted when PIM447 was added. This suggests that combination therapy, in which a Pim kinases inhibitor is added to ALL treatment, cannot only aid in reducing ALL leukemia cells, but also could be beneficial in reducing the MRD burden.

**Table 4:** Pim kinase inhibitors used in preclinical and clinical (bold) trials for ALL

Acute Lymphoblastic Leukemia	Target	Type	Clinical Trials
AZD1208	pan-Pim	Single	Yes
AZD1208 + Ponatinib or Dasatinib	pan-Pim + TKI	Dual-therapy	–
AZD1208 + Ponatinib + Z-VAD-FMK	pan-Pim + TKI+ pan-caspase	Triple-therapy	–
AZD1208 + Sabutoclax	pan-Pim + BCL2	Dual-therapy	–
AZD1208 + inLCK	pan-Pim + LCK	Dual-therapy	–
LGB321	pan-Pim	Single	Preclinical
PIM447	Pan-Pim	Single	Yes
Smi-4a	Pim1	Single	Preclinical
Smi-4a + U0126	pan-Pim + MEK1/2	Dual	–
TP-3654	Pan-Pim	Single	Preclinical
TP-3654 and dexamethasone	Pan-Pim and glucocorticoid therapy	Dual-therapy	–

Several human clinical trials of Pim inhibitors to treat MM are underway (Table 5). MM patients were included in the clinical trials with Uzansertib that were discussed with the AML patients above. Phase I clinical trials with PIM447 in relapse/refractory MM are completed; however, results have not been officially published. In one trial of 79 patients with at least four previous treatments, the partial response rate (or better) was 8.9% with a clinical benefit rate of 25.3% [169]. PIM447 was well tolerated even with adverse events that comprised thrombocytopenia and anemia. A smaller study, comprised of 15 Japanese patients with relapsed or refractory MM, showed an overall response rate of 15.4% and a similar clinical benefit rate of 23.1%, with at least 2 patients achieving partial responses [170]. Thrombocytopenia, anemia, and leukopenia were also observed at high rates. These results are highly significant to MM therapy, considering these patients are often refractory to other therapies. As is the case with other leukemias, these studies suggest that Pim inhibitors can be used and are relatively well tolerated in MM patients but may require dual or multi-inhibitor combination therapies. In preclinical studies both Pim kinases inhibitors SGI-1776 and LGB321 inhibit MM cells in mouse xenograph models [54, 171]. LGB321 could combine with BKM12 (inPI3K), with subsequent loss of mTOR activity. The underlying mechanism was related to inhibition of Pim-2 phosphorylation of TSC2 and activation of mTOR activity in MM cells [54]. Additional studies showed that the Pim kinase inhibitor Smi-16a inhibits MM cell viability and demonstrated synergistic effect when combined with doxorubicin [172]. Dual and triple drugs that contain Pim kinase inhibitors, along with PI3K and/or mTOR inhibitors have also been effective in MM cells [172]. Based on these observations it is tempting to speculate that combination treatment used to treat AML patients could be effective for MM patients. The treatments developed to target FLT3, CXCR4, and the PI3K pathway could work in conjunction with Pim inhibitors in MM, given the similarity in oncogenic pathways found in both these cancers. Furthermore, Pim kinase inhibition also relieve symptoms of osteolytic bone lesions often seen in MM patients and in pre-clinical trials, treatment of MM cells with Smi-16a or PIM447 prevents MM tumor growth in mice and suppresses myeloma-induced bone destruction [109, 111]. Finally, targeting miRNAs that regulate Pim kinases may represent future solutions for the treatment of MM. The proteasomal inhibitor, MLN2238, has been used in clinical trials and has been found to positively regulate miR-33a expression [171]. miR-33a directly regulates Pim-1 and MM cells treated either with miR-33a or MLN2238 suffer reduced Pim-1 signaling and cell viability.

**Table 5:** Pim kinase inhibitors used in preclinical and clinical (bold) trials for MM

Multiple Myeloma	Target	Type	Clinical Trials	
IBL-202	Pim/PI3K	Double inhibitor (PI3K, Pim) Monotherapy	Preclinical	–
IBL-301	Pim/PI3K/mTOR	Triple inhibitor (PI3K, mTOR, Pim) Monotherapy	Preclinical	–
**INCB053914 + pomalidomide + dexamethasone**	**pan-Pim + thalidomide analogue + corticosteroid**	**Triple-therapy**	**MM**	**NCT04355039**
JP11646	Pim2	Single	Preclinical	–
LGB321	pan-Pim	Single	Preclinical	–
LGB321 + BKM120	pan-Pim + PI3K	Dual-therapy	Preclinical	–
**PIM447**	**pan-Pim**	**Single**	**MM**	**NCT01456689**
			**MM**	**NCT02144038**
SGI-1776	pan-Pim	Single	Yes	–
Smi-16a	Pim1/Pim2	Single	Preclinical	–
Smi-16a + doxorubicin	Pim1/Pim2 + topoisomerase	Dual-therapy	–	–
**Uzansertib**	**pan-Pim**	**Single**	**MM**	**NCT04355039**

One of the first Pim kinases Phase I clinical trials included lymphoma patients (Table 6). As discussed above, Phase I clinical trials with SGI-1776 on relapsed/refractory NHL patients, showed proof of principle – that Pim kinase inhibition could be effective in treatment of lymphoma, but was terminated due to adverse cardiac events. Preclinical trials with SGI-1776 and Pim kinase inhibitors in MCL cells have also been successful, targeting transcription and translation in these cells [90, 173]. Preclinical and clinical data suggests that Pim kinase inhibition may facilitate to overcome the resistance associated with long-term chemotherapy. In fact, in Phase I trials of patients with recurrent NHL that has disseminated to the central nervous system (CNS), high Pim-2 expression was a marker for resistance to rituximab monotherapy [174]. In studies of MCL patients given high dose therapy, stem cell transplantation, and rituximab; Pim-1 expression also served as a predictive marker of poor outcome [120]. The CDK/Pim/FLT3 inhibitor, ETH-155008, is currently being assessed in refractory/relapsed B-cell, NHL patients as well as CLL and SLL patients. Clinical trials have also been conducted with DLBCL patients. Uzansertib has been used in Phase I clinical trials with relapsed/refractory DLBCL patient samples; however, results have not been reported. Incyte has actively recruited patients to test dual-therapy, Uzansertib with a PI3K inhibitor (Parsaclisib). This was based on preclinical data showing the effectiveness of Uzansertib along with Parsaclisib or JAK inhibitors (itacitinib or ruxolitinib), or cytarabine (a common chemotherapy reagent) in DLBCL xenografts [143]. While clinical trials have not been initiated for many of the NHLs and cHLs, preclinical data is very promising. As discussed above, preclinical xenograft models of ATL with AZD1208 led to reduced tumor burden, while in vitro treatment of PTCLs and cHLs with Pim inhibitors and/or led to reduced proliferation. LGB321 was also successful in reducing tumor burden in CLL xenografts. Similar to other conclusions, tumor burden was further reduced when the Pim kinase inhibitor was given as dual therapy with ibrutinib, an inhibitor of Bruton’s tyrosine kinase (BTK) [175]. Treatment with AZD1208 and ibrutinib was also successful in DLBCL cells [176].

**Table 6:** Pim kinase inhibitors used in preclinical and clinical (bold) trials for cHL and NHL

	Pim Inhibitor	Target	Type	Clinical Trials	
cHL	SEL24/MEN1703	PIM/FLT3	Double inhibitor (FLT3, Pim) Monotherapy	Yes	–
	Pim Inhibitor + suberoylanilide hydroxamic acid (SAHA) or sodium butyrate (SB	Pim + Histone deacetylase inhibitors	Dual-therapy	Preclinical	–
DLBCL	AZD1208 + ibrutinib	pan-Pim + BTK	Dual-therapy	Preclinical	–
	**ETH-155008**	**Pim3-CDK4/6(FLT3)**	**Triple inhibitor (FLT3, Pim-3 and CDK4/6) Monotherapy**	**DLBCL**	**NCT04840784**
	**Uzansertib**	**pan-Pim**	**Single**	**DLBCL**	**NCT03688152**
	**Uzansertib + Parsaclisib**	**pan-Pim + PI3K**	**Dual-therapy**	**DLBCL**	**Incyte**
	Uzansertib + Itacitinib (or ruxolitinib)	pan-Pim + JAK	Dual-therapy	Preclinical	–
	Uzansertib + cytarabine	pan-Pim + Chemotherapy	Dual-therapy	Preclinical	–
PTCL	ETP-39010	pan-Pim	Single	Preclinical	–
	ETP-47551	pan-Pim	Single	Preclinical	–
MCL	**SGI-1776**	**PIM1**	**Single**	**MCL**	**NCT01239108**
CLL/SLL	K00135	Pim1/Pim2	Single	Preclinical	–
	K00486	Pim1/Pim2	Single	Preclinical	–
	A47	pan-Pim	Single	Preclinical	–
	**ETH-155008**	**Pim3-CDK4/6 (FLT3)**	**Triple inhibitor (FLT3, Pim-3 and CDK4/6) Monotherapy**	**CLL/SLL**	**NCT04840784**
	SGI-1776	PIM1	Single	Yes	–
	SEL24/MEN1703	PIM/FLT3	Double inhibitor (FLT3, Pim) Monotherapy	Yes	–
	LGB321	pan-Pim	Single	Preclinical	–
	LGB321 + ibrutinib	pan-Pim + BTK	Dual-therapy	Preclinical	–
ATL	Smi-16a	Pim1/Pim2	Single	Preclinical	–
	AZD1208	pan-Pim	Single	Yes	–

JAK2 mutations, especially V617F, play a critical role in MPN pathogenesis and drug resistance; however, JAK2 inhibitor monotherapy is largely ineffective in achieving consistent remission in MPN patients carrying these mutations. Resistance to JAK2 inhibitors has been shown in MPN cells carrying JAK2 mutations and in CML patients harboring the BCR-ABL translocation [177]. Because tumor cells rely upon constitutive activation of STAT5, ERK-1/2 (Extracellular signal-regulated kinase 2), and AKT signaling pathways, targeting down-stream JAK2 targets, such as Pim kinases may have significant benefits in preventing JAK2 inhibitor resistance (Table 7). Indeed, the fact that JAK2 chemo resistant cells harboring the V617F mutation display high expression of Pim kinases, compared to sensitive cell lines, suggests a role of Pim kinases in drug resistance [178]. Pan-Pim kinase inhibitors, such as AZD1208, Uzansertib, and SGI-1447 have been shown to be effective in ex vivo MPN patient samples, when combined with JAK inhibitors (ruxolitinib, AZ960, TG101209, and SAR302503) [133, 178–181] (Fig. 6). Unlike other Pim inhibitors, preclinical models with single therapy, TP-3654 have shown efficacy, even over JAK2 inhibitors (ruxolitinib) in treating fibrosis MF in vivo models [135]. Furthermore, the combination of TP-3654 with ruxolitinib led to greater reductions in MPN cells and fibrosis. Preclinical data is also promising for the combination of PIM447, ruxolitinib, and the CDK4/6 inhibitor, LEE011, in JAK2 V617F and MPL515L in vivo MF models, with longer survival and no additive toxicity seen [182].

**Table 7:** Pim kinase inhibitors used in preclinical and clinical (bold) trials for MPNs

	Pim Inhibitor	Target	Type	Clinical Trials	
MPNs	AZD1208	pan-Pim	Single	Yes	–
	AZD1208 + Ruxolitinib	pan-Pim + JAK	Dual-therapy	Preclinical	–
	SGI-1776	Pim1/Pim2	Single	Yes	–
	INCB053914 + Ruxolitinib	pan-Pim + JAK	Dual-therapy	Preclinical	–
	**PIM447**	**pan-Pim**	**Single**	**MF**	**NCT02370706**
	PIM447 + Ruxolitinib + LEE011	pan-Pim + JAK + CDK4/6	Triple-therapy	Preclinical	–
	**TP-3654**	**pan-Pim**	**Single**	**MF**	**NCT04176198**
	TP-3654 + Ruxolitinib	pan-Pim + JAK	Dual-therapy	Preclinical	–
	Pim inhibitor (PIM447) + SAR302503 (TG101348)	pan-Pim + JAK	Dual-therapy	Preclinical	–
	**Uzansertib**	**pan-Pim**	**Single**	**MF**	**NCT02587598**
CML	Smi-4a	Pim1	Single	Preclinical	–
	AZD1208	pan-Pim	Single	Yes	–
	AZD1208 + imatinib mesylate	pan-Pim + TKI	Dual-therapy	No	–
	SGI-1776	Pim1		Yes	–
	SGI-1776 + imatinib mesylate	pan-Pim + TKI	Dual-therapy	No	–

In BCR-ABL carrying CML cells Pim inhibitors influenced cell viability and proliferation by targeting GSK3-β (glycogen synthase kinase 3 beta), mTOR, translation, and MYC/Mcl-1 [183, 184]. Chemoresistance of cancer stem cells represent a significant challenge and CML stem cells are inherently resistant to imatinib mesylate, a standard therapy for CML. Part of this resistance is due to high levels of Pim-2, which phosphorylates and inactivates proapoptotic factor, BAD. While imatinib mesylate was effective in treating CML stem cells with high Pim-2 expression induced through STAT5; imatinib mesylate resistant cells continued to exist due to activation of Pim-2 through an alternative, STAT4 dependent pathway [185]. To overcome this block in imatinib mesylate CML therapy, mono Pim kinase inhibitor or dual therapy with imatinib mesylate has been used [183, 184]. Treatment with SGI-1447 or AZD1208, along with imatinib mesylate, has a suppressive effect on Ph + primitive, imatinib mesylate sensitive, leukemic progenitor cells and imatinib mesylate insensitive, CML stem cells from CML patients, respectively [183, 185]. This suggests (like other lymphoproliferative cancers) that effective inhibition of Pim kinase activity can be a very successful treatment option to reduce disease burden and/or overcome resistance to current therapies.

## Conclusion

Clinical trials using first generation Pim inhibitors have failed to significantly reduce disease burden or alleviate chemoresistance in patients. In addition, higher doses of Pim inhibitors may have off-target effects and can lead to adverse effects, including cardiac events, gastrointestinal issues, febrile neutropenia, and rash, among others. Newer, highly specific Pim kinase inhibitors, such as PIM447, Uzansertib, and TP-3654 can overcome these problems, allowing Pim targeting at lower concentrations. Another option is to directly target Pim kinases through either monoclonal Pim antibody therapy or by indirectly targeting Pim kinase regulators. Pim-1 was found on the cell surface of some leukemic cells, that could be targeted, with loss of proliferation, by monoclonal Pim-1 (mPim-1) therapy [186]. In addition, in vivo prostate cancer models treated with mPim-1, led to tumor growth reductions [187]. These early, pre-clinical trials suggest mPim-1 therapy may work; however, whether redundancy with other Pim kinases would reduce in vivo efficacy remains to be seen. Previous studies in MM and CML suggest that Pim kinases are subject to 3’UTR mediated down-regulation by miRNAs through miR-33a and miR-328, respectively. Pim-1 and Pim-3 are both regulated by miR-33a [188, 189]; and in various tissues and cell lines, the 3’UTR of Pim-1 is also targeted for inhibition by miR-206 [190], miR-214 [122], miR-370 [191], miR-486-5p, [192] miR-328 [137, 193], miR-124 [194], miR-144 [195], miR-542-3p [196], miR-101-3p [197]. Pim-2 is targeted by miR-26-5p [198] and miR-135-3p [199], while Pim-3 can also be targeted by miR-506 [200]. Targeting miRNAs directly or indirectly, such as is the case with MLN2238 in MM, may be a future direction for therapy.

Effective strategies to treat leukemia/lymphoma will likely incorporate dual or combination therapy that includes a pan-Pim inhibitor. Numerous pre-clinical studies have demonstrated increased efficacy when Pim inhibitors are part of a dual therapy regimen along with JAK inhibitors. Pim kinases are elevated by common protein tyrosine kinase receptors that are deregulated in most lymphoproliferative disorders, therefore targeting Pim and the JAK/STAT pathway is a very interesting option. This would allow reduced Pim inhibitor dosing, while possibly preventing JAK inhibitor resistance. In addition, several JAK inhibitors are part of common chemotherapy regimens and have good safety profiles, including ruxolitinib for lymphoproliferative disorders and, tofacitinib, baricitinib, and upadacitinib, for rheumatoid arthritis that have already gained FDA approval. The dual Pim-CDK4/6 inhibitor, abemaciclib, and multi-inhibitors, Pim-3-CDK4/6-FLT3, ETH-155008, and PIM-FLT3, SEL24/MEN1703 show promise and are currently undergoing clinical trials. Another strategy is to pair pan-Pim inhibitors with inhibitors of the PI3K/AKT/mTOR pathway, for which Pim kinases show parallel functions. Clinical trials are already underway or completed for drugs like, AKT inhibitor, MK-2206, for CLL/SLL and relapse/refractory leukemias or lymphomas, while mTOR inhibitors sirolimus (rapamycin) and everolimus are being used in clinical trials. Dual therapy with Bcl-2 or Mcl-1 antagonists with pan-Pim inhibition may also prove useful, as evidenced by results from preclinical data in ALL with sabutoclax (in-Bcl-2). Future studies are still needed to delegate the specific roles of individual Pim isoforms, their downstream targets, and how Pims are regulated to refine therapeutic options more precisely. However, the sheer number of pre-clinical and clinical trials being conducted with Pim inhibitors demonstrates the clinical significance of targeting the Pim pathway in lymphoproliferative disorders and solid tumors.

## Abbreviations

4E-BP1: Eukaryotic Translation Initiation Factor 4E Binding Protein 1
ABC-DLBCL: Activated B-cell-like DLBCL
ABCB1/2 (BCRP): ATP Binding Cassette Subfamily G Member
ABC transporters: ATP-binding cassette transporters
AIDS-NHL: AIDS-related non-Hodgkin lymphoma
AKT: AKT serine/threonine kinase 1
ALL: Acute lymphoblastic leukemia
ALT: Alanine aminotransferase
AML: Acute myeloid leukemia
APL: Acute promyelocytic leukemia
AR: Androgen receptor
ASK1: Apoptosis signaling kinase 1
AST: Aspartate aminotransferase
ATLL: Adult T-cell leukemia/lymphoma
B-ALL: B-lymphoblastic leukemia ALL
BAD: Bcl2 associated antagonist of cell death
Bcl-2: BCL2 Apoptosis Regulator
Bim: BCL2 Like 11
Bmi-1: B Lymphoma Mo-MLV Insertion Region 1 Homolog
BCR/ABL-Ph+: Breakpoint cluster region/Proto-oncogene tyrosine-protein kinase ABL1
BL: Burkitt’s lymphoma
C-MyB and A-Myb: Myb Proto-Oncogene
C-Myc/N-Myc: C/N-MYC Proto-Oncogene
C-TAK1: Protein kinase Cdc25 C-associated kinase 1
CALR: Calreticulin
CDC25A/C: Cell Division Cycle 25A and C
cHL: Hodgkin’s lymphoma
CLL: Chronic lymphocytic leukemia
Cot: MAP3K8/Mitogen-Activated Protein Kinase Kinase Kinase 8
CML: Chronic myeloid leukemia
CNS: Central nervous system
CTL: Cutaneous T-cell lymphoma
CXCR4: C-X-C Motif Chemokine Receptor 4
eIF4B: Eukaryotic Translation Initiation Factor 4B
ERK-1/2: Extracellular signal-regulated kinase 2
ET: Essential thrombocythemia
ETP-ALL: Early T-cell precursor ALL
FL: Follicular lymphoma
FLT3: Fms-like tyrosine kinase 3
FoxO1a and FoxO3a: Forkhead box O1a and 3a
GC: Germinal center
H3: Histone H3
H4/PDGFβR: D10S170/Platelet derived growth factor receptor beta
HOXA9: Homeobox A9
HP1γ: Heterochromatin-associated protein 1 gamma
HTLV-1: Human T-cell leukemia virus type-1
IGV: Immunoglobulin variable
IKKα/β: I-Kappa-B Kinase alpha/beta
IL-2: Interleukin-2
IL-7Rα: Interleukin-7
ITD: Internal tandem duplication
JAK: Janus kinase
LKB1: Serine/Threonine Kinase 11
LYL1: Lymphoblastic leukemia derived sequence 1
M-MuLV: Moloney-murine leukemia virus
mPim-1: Monoclonal Pim-1
mTOR: Mammalian target of rapamycin
MALT: Transformed mucosa-associated lymphoid tissue lymphoma
MCL: Mantle cell lymphoma
MCL1: BCL2 family apoptosis regulator
MDM2: MDM2 Proto-Oncogene
MDS: Myelodysplastic syndromes
MEK1/2: Mitogen-activated protein kinase kinase ½
MF: Myelofibrosis
MM: Multiple myeloma
MPL: Thrombopoietin receptor
MYD88: Myeloid Differentiation Primary Response Protein MyD88
NF-κB: NF-Kappa-B transcription factor
NFATC1: Nuclear factor of activated T-cells 1
NKX3.1: NK3 Homeobox 1
NHL: Non-Hodgkin’s lymphoma
NLPHL: Nodular lymphocyte-predominant HL
NMZL: Nodal marginal zone lymphoma
Notch-1/3: Notch receptor 1 and 3
NuMA: Nuclear Mitotic Apparatus
NUP214-ABL1: Nucleoporin Nup214-ABL1
p21CIP1/WAF1: CDKN1A/cyclin Dependent Kinase Inhibitor 1A
p27KIP1: CDKN1B/cyclin Dependent Kinase Inhibitor 1B
p70S6K: Ribosomal protein S6 kinase beta-1
p100: Nuclear Factor Kappa B Subunit 2
Pal-1/Gfi-1: Growth Factor Independent 1 Transcriptional Repressor
Pax-5: Paired Box 5
PBMCs: Peripheral blood mononuclear cells
PCNSL: Primary central nervous system lymphoma
PI3K: PI3-kinase
Pim: Provirus Integration site for Moloney leukemia virus
PMLBCL: Primary mediastinal large B-cell lymphoma
PRAS40: AKT1 Substrate 1
PTCL: Peripheral T-cell lymphoma
PTPN2: Protein tyrosine phosphatase non-receptor type 2
PV: Polycythemia vera
RARα: Retinoic acid receptor alpha
RhoH: Ras homolog family member H
Runx2: RUNX Family Transcription Factor 2
SAHA: Suberoylanilide hydroxamic acid
SHA: Somatic hypermutation activity
SLL: Small lymphocytic lymphoma
SOCS-1/3: Suppressor of Cytokine Signaling 1/3
STAT: Signal transducer and activator of transcription
TCRβ-PIM1: T-cell receptor beta locus/PIM1
TEL/AML1: Transcription Factor ETV6/RUNX Family Transcription Factor 1
TEL/TRKC: TEL/tyrosine kinase receptor C
TFF: Trefoil Factor
TSC2: TSC Complex Subunit 2
UTR: Untranslated region
WHO: World Health Organization
